# Amyloid Fibrils Formed by Short Prion-Inspired Peptides Are Metalloenzymes

**DOI:** 10.1021/acsnano.3c04164

**Published:** 2023-08-30

**Authors:** Susanna Navarro, Marta Díaz-Caballero, Francesca Peccati, Lorena Roldán-Martín, Mariona Sodupe, Salvador Ventura

**Affiliations:** † Institut de Biotecnologia i de Biomedicina (IBB) and Departament de Bioquímica i Biologia Molecular, Universitat Autònoma de Barcelona, 08193 Bellaterra (Barcelona), Spain; ‡ Basque Research and Technology Alliance (BRTA), Center for Cooperative Research in Biosciences (CIC bioGUNE), 48160 Derio, Spain; § Departament de Química, Universitat Autònoma de Barcelona, 08193 Bellaterra (Barcelona), Spain

**Keywords:** peptides, amyloid fibrils, self-assembly, nanoenzymes, biocatalytic nanomaterials, bioremediation

## Abstract

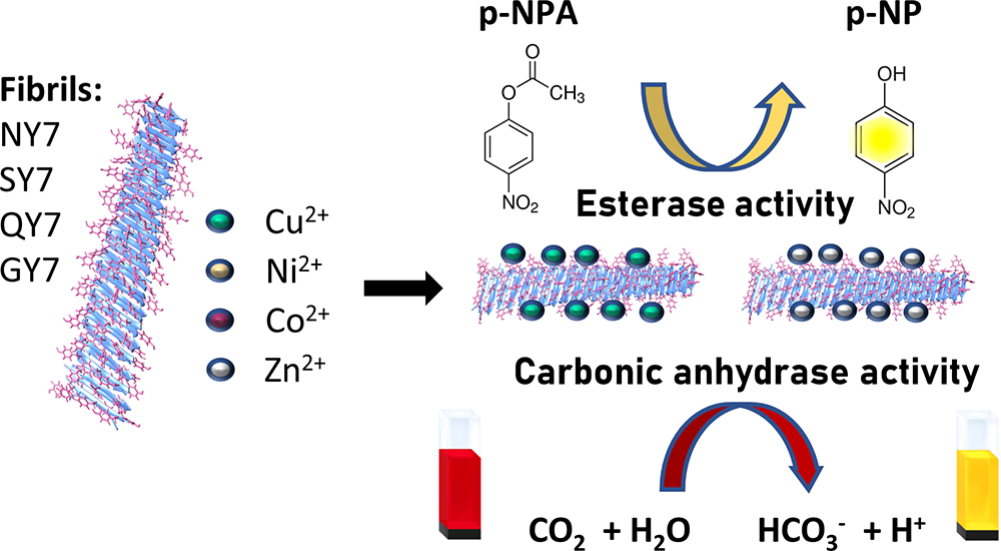

Enzymes typically fold into defined 3D protein structures exhibiting a high catalytic efficiency and selectivity. It has been proposed that the earliest enzymes may have arisen from the self-assembly of short peptides into supramolecular amyloid-like structures. Several artificial amyloids have been shown to display catalytic activity while offering advantages over natural enzymes in terms of modularity, flexibility, stability, and reusability. Hydrolases, especially esterases, are the most common artificial amyloid-like nanozymes with some reported to act as carbonic anhydrases (CA). Their hydrolytic activity is often dependent on the binding of metallic cofactors through a coordination triad composed of His residues in the β-strands, which mimic the arrangement found in natural metalloenzymes. Tyr residues contribute to the coordination of metal ions in the active center of metalloproteins; however, their use has been mostly neglected in the design of metal-containing amyloid-based nanozymes. We recently reported that four different polar prion-inspired heptapeptides spontaneously self-assembled into amyloid fibrils. Their sequences lack His but contain three alternate Tyr residues exposed to solvent. We combine experiments and simulations to demonstrate that the amyloid fibrils formed by these peptides can efficiently coordinate and retain different divalent metal cations, functioning as both metal scavengers and nanozymes. The metallized fibrils exhibit esterase and CA activities without the need for a histidine triad. These findings highlight the functional versatility of prion-inspired peptide assemblies and provide a new sequential context for the creation of artificial metalloenzymes. Furthermore, our data support amyloid-like structures acting as ancestral catalysts at the origin of life.

## Introduction

Enzymes are the molecular entities responsible for most biological reactions. Modern enzymes are typically proteins of at least 100 residues that fold into defined 3D native structures showcasing high catalytic efficiency and selectivity.^1−3^

It has been hypothesized that the earliest enzymes on Earth might have arisen from the spontaneous self-assembly of short peptides to form supramolecular structures.^4−6^ Specifically, it has been suggested that amyloids may have acted as the scaffold supporting primitive catalytic activities.^7,8^ As amyloids use amino acid sequences as their building blocks, and their assembly is driven and sustained by noncovalent interactions, they share similarities with natural proteins.^9^ In favor of this hypothesis, several artificial amyloids formed by short, and often repetitive, amino acid sequences have been shown to act as enzyme mimetics.^10−14^ Although, in general terms, they are catalytically less efficient than modern enzymes,^15^ the modularity, flexible design, stability, and reusability of these nanozymes endow them with advantages,^16,17^ relative to their natural counterparts, including lower synthesis and modification costs.

About one-third of modern enzymes are metalloenzymes that exploit the coordination of metal ions to catalyze some of the most complex chemical reactions in nature.^18^ Among them, many hydrolases depend on the coordination of divalent metallic cations, frequently Zn^2+^,^19^ to exert their activities, like in carbonic anhydrases (CAs). Coordination of Zn^2+^ lowers the p*K*_a_ of a bound water, facilitating the formation of a hydroxide for nucleophilic attack of the substrate.^20^ CAs are ubiquitous and highly efficient metalloenzymes that participate in distinct biological reactions, including the capture of CO_2_ in erythrocytes via its hydration into bicarbonate (HCO_3_^–^), with the production of protons (H^+^).^21,22^ This CO_2_ sequestration ability, together with their esterase activity, makes CA enzymes of high interest in biotechnology.^23^

Among artificial amyloid-like nanozymes, the most common are those exhibiting hydrolytic activity, especially esterases,^8,10,24−29^ with some of them reported to act as CAs.^24,27,30−32^ In a large majority of cases, their function is strictly dependent on the presence of a metal cation, with Zn^2+^ being used on most occasions. In natural enzymes, the Zn^2+^ binding site often encompasses two His residues projecting from the same β-strand and a third His ligand from an adjacent strand.^33^ This elementary interstrand 3-His metal-coordinating site is typically found in CAs.^34^ This β-sheet architecture makes the amyloid fold ideal for building up artificial esterases and CAs,^10,24^ although unlike in modern enzymes, the fibrils’ catalytic sites are surface-exposed and not buried in the protein core.

All metal-dependent amyloid-based esterases reported so far rely strictly on coordinating metal ions by His residues, although they differ in the placement of these residues in the peptide building block. In some cases, His are intercalated with hydrophobic residues that provide the driving force for the assembly,^8,24,25^ whereas on other occasions, the catalytic and assembly moieties are segregated at the two extremes of the sequence.^27,31,32^ In many instances, the presence of the metal in the assembly solution is a requirement not only for the activity but also to attain a stable fibrillar architecture.^8,24^

Different studies have shown that l-Tyr can bind to transition metals;^35^ most coordinations occur at the amino nitrogen or carboxylic oxygen, but the phenolic oxygen can also be involved. Indeed, Tyr residues contribute to coordinate metal ions in the active centers of proteins like ferritin,^36^ cytochrome oxidase,^37^ lytic polysaccharide monooxygenases,^38^ and galactose oxidase,^39^ often through the phenolic hydroxyl group. Nevertheless, despite this evidence, the use of Tyr has been mostly neglected in the design of metal-containing amyloid-based nanozymes.

Recently, we have reported a set of low-complexity, polar heptapeptides inspired by prion-like domains’ composition, which spontaneously self-assemble into highly stable supramolecular amyloid fibrils endowed with redox activity.^40,41^ Here, we characterized the interaction of these His-devoid but Tyr-rich peptides with divalent metal cations. Once assembled, these peptides bind and become decorated with Co^2+^, Cu^2+^, Ni^2+^, and Zn^2+^. The metallized fibrils act as nanozymes exhibiting esterase and CA activities without requiring a histidine triad. Overall, our data support the hypothesis that amyloid-like structures may have played a catalytic role in the emergence of life while demonstrating the functional versatility of prion-inspired peptide fibrils.

## Results and Discussion

### 1 Prion-Inspired Fibrils Retain Cu^2+^, Ni^2+^ Co^2+^, and Zn^2+^ Divalent Metal Cations

We have shown that seven-residue-long polar sequences inspired in the amino acid composition of prion-like domains, NYNYNYN (NY7), QYQYQYQ (QY7), SYSYSYS (SY7), and GYGYGYG (GY7), spontaneously self-assemble into amyloid-like fibrillar structures endowed with redox activity.^32^ Based on the fibril models we generated previously,^40^ the assemblies expose multiple phenolic hydroxyls to the solvent (Figure S1), suggesting the possibility that the fibrils could bind to divalent metal cations.

**Figure 1:**
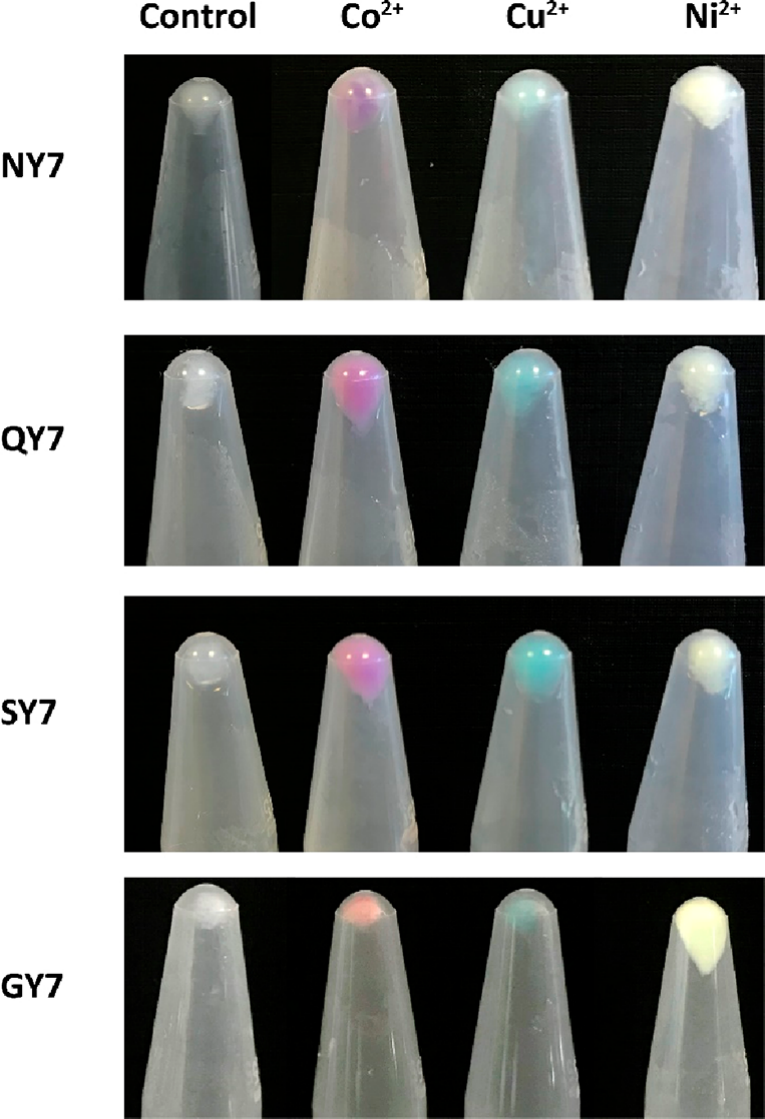
Pelleted fibrils containing divalent metal cations Co^2**+**^, Cu^2**+**^, and Ni^2**+**^. Pictures corresponding to control pelleted fibrils NY7, QY7, SY7, and GY7 incubated only with buffer (translucid) and incubated with Co^2+^ (red), Cu^2+^ (blue), and Ni^2+^ (yellow), respectively.

To elucidate whether NY7, QY7, SY7, and GY7 fibrils could bind Cu^2+^, Ni^2+^, Co^2+^, and Zn^2+^, they were incubated in the presence of different concentrations of CuCl_2_, NiSO_4_, CoCl_2_, and ZnCl_2_. Their retention capacity was determined by exploiting the metal colorations. Upon centrifugation, the fibrils rendered colored pellets that were visible to the naked eye (Figure 1), indicative of the binding of metal cations. Cu^2+^-incubated fibrils were blue, Ni^2+^ yellowish, and Co^2+^ purple for NY7, QY7, and SY7 and orangish for GY7, which might reflect different Co^2+^ oxidation states. In the case of Zn^2+^, detection was not possible by a simple visual inspection because the solution was transparent.

To confirm the ability of the fibrils to sequester Cu^2+^, Ni^2+^, and Co^2+^ we determined the metals’ molar extinction coefficients (ε) in solution (ε_Ni2+393 nm_ = 48 mM^–1^·cm^–1^, ε_Co2+512 nm_ = 60 mM^–1^·cm^–1^, and ε_Cu2+235 nm_ = 189 mM^–1^·cm^–1^) (Figure S2). For Zn^2+^_,_ the absorbance maximum was below 200 nm, limiting its spectroscopic detection. Accordingly, Zincon dye, which binds Zn^2+^ with high affinity, presenting an absorbance maximum at 628 nm, was used (ε_Zincon-Zn2+628 nm_ = 228 mM^–1^·cm^–1^). The retention percentages were calculated by comparing the absorbance of solutions of each divalent cation without fibrils with those measured in the supernatants of fibril-containing solutions after centrifugation using the Lambert–Beer equation. The assayed concentration range was adjusted for the different divalent metal cations according to their spectrophotometric properties. Figure S3 illustrates how all the fibrils can scavenge Cu^2+^, Ni^2+^, Co^2+^, and Zn^2+^ from the solution, with the percentage of retention depending on the nature of the cation and the heptapeptide that conforms to the fibrils. While these qualitative spectrophotometric measurements confirmed the fibrils’ metal retention ability, additional assays were required to quantify the binding more accurately.

### 2 Quantification of Metal Coordination by Prion-Inspired Fibrils

To quantify the metal retention capacity of our prion-inspired fibrils, we used inductively coupled plasma optic emission spectroscopy (ICP-OES). First, we calculated the concentration of heptapeptides in both the soluble and insoluble fractions of the self-assembled reactions. Next, based on the divalent metal cations’ quantification by ICP-OES in centrifuged fibrils, after their incubation with 200 mM Cu^2+^, Ni^2+^, Co^2+^, and Zn^2+^ (Table S1), we calculated the specific retention capacity for each metal/peptide combination (Table 1).

**Table 1:** NY7, QY7, SY7, and GY7 Fibrils’ Divalent Cation Retention Capacity^a^

	cation	NY7	QY7	SY7	GY7
retention capacity (μmol cation/ μmol peptide)	Cu^2+^	34.1 ± 8.4	69.2 ± 12.5	103.5 ± 9.3	58.1 ± 2.7
	Ni^2+^	15.5 ± 1.2	35.8 ± 1.3	72.8 ± 17.3	15.3 ± 11.0
	Co^2+^	38.2 ± 4.7	68.1 ± 3.0	124.6 ± 24.7	46.2 ± 6.1
	Zn^2+^	35.0 ± 4.6	62.8 ± 9.8	92.5 ± 26.3	36.9 ± 10.0

a Divalent cations Cu^2+^, Ni^2+^, Co^2+^, and Zn^2+^ retention capacity was calculated in μmol cation/μmol peptide. Values indicate the mean of duplicates from two independent experiments (± standard deviation).

The results enabled us to rank the four fibrils according to their cation retention. Overall, SY7 fibrils displayed the highest retention capacity, followed by QY7, while GY7 and NY7 showed lower retention. The binding capacity of all fibrils was found to be remarkable, with each μmol of peptide retaining at least 15 μmol of the corresponding divalent metallic cation and up to 125 μmol for some metal/peptide combinations. These results demonstrate that the prion-inspired amyloid scaffolds function as effective metallic traps, as observed in previous studies for β-lactoglobulin fibrils, which, in contrast to our assemblies, were formed upon extensive denaturation at pH 2.0 and 90 °C.^42,43^

### 3 Docking of Divalent Metallic Metals on Prion-Inspired Fibrils

**Figure 2:**
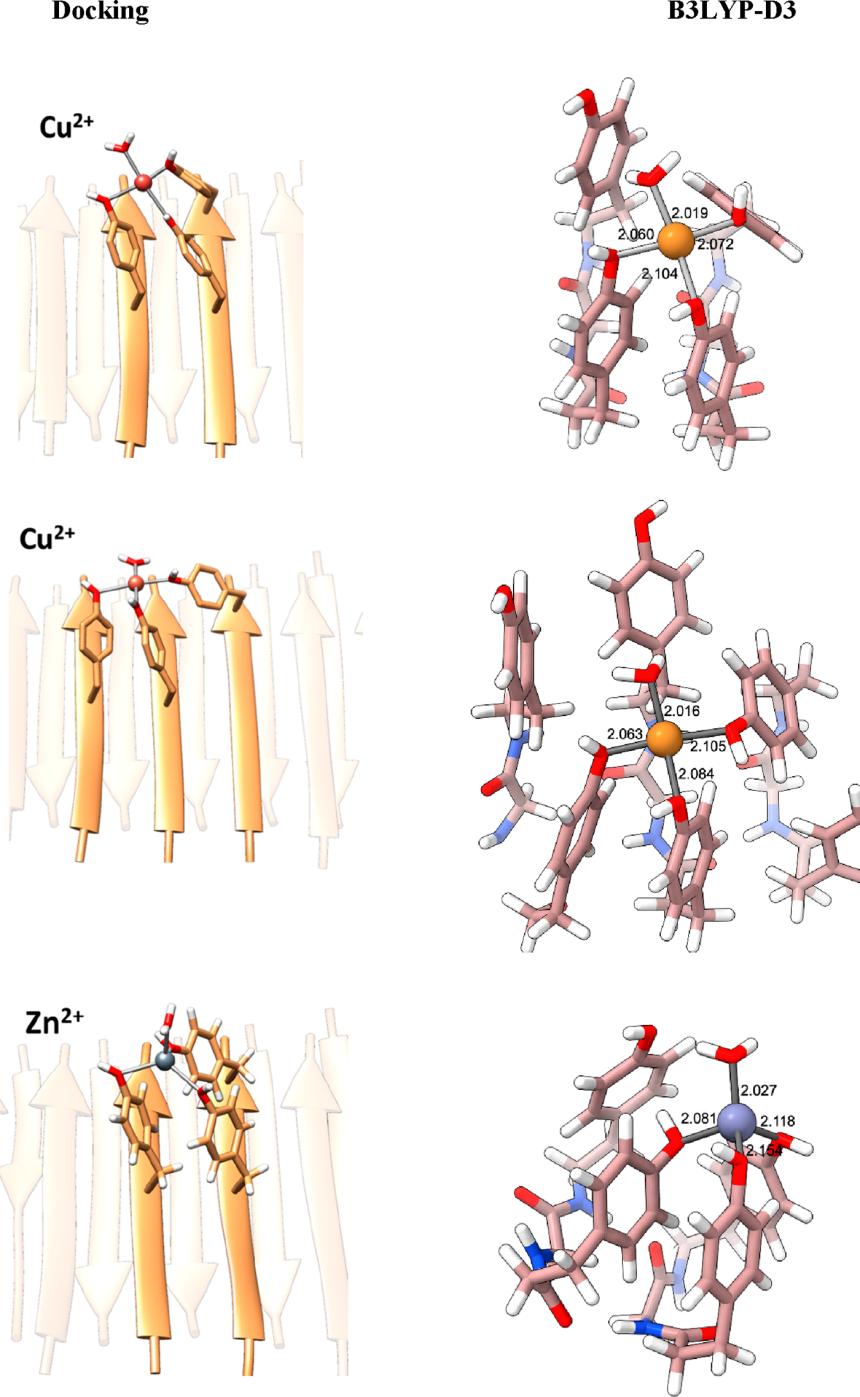
Docking of metals on fibrils. Cu^2+^ and Zn^2+^ (II) plausible binding sites in SY7 from docking simulations, in square planar and tetrahedral geometry, respectively, and B3LYP-D3-optimized geometries. Distances are in Å.

To identify how metal ions can coordinate to the solvent-exposed Tyr residues in the fibrils, we selected those with the highest specific binding capacity (SY7) and the biologically relevant Cu^2+^ and Zn^2+^ cations. We performed docking calculations on the fibril models previously built by us^40^ and run electronic structure DFT(B3LYP-D3) calculations to evaluate whether these binding sites are stable or not. According to the coordination properties of these metal ions, we considered a square planar environment for Cu^2+^ (3d^9^) and a tetrahedral one for Zn^2+^ (3d^10^).^44^ Docking simulations indicate that the maximum number of Tyr residues that can simultaneously coordinate to either Cu^2+^ or Zn^2+^ is three (Figure 2). The vacant site is thus filled with a water molecule.

For Cu^2+^ two different sets of results, with a coordination sphere formed by Tyr from either two or three (Figure 2) different strands, were obtained. For Zn^2+^, however, in a tetrahedral environment, only the coordination of Tyr from two different strands was encountered. Quantum chemical calculations at the B3LYP-D3 level of theory confirmed that these binding modes are stable, metal–oxygen(Tyr) bonding distances being about 2.1 Å, similar to the values 2.2–2.3 Å identified in metalloproteins.^44^ Interaction of metal cations with the π system of two phenolic moieties was also explored, but calculations indicate that such coordination (Figure S4) is significantly less stable (17 kcal mol^–1^ for Cu^2+^ and 42 kcal mol^–1^ for Zn^2+^) than with the oxygens of Tyr shown in Figure 2.

To further assess the availability of Tyr side chains for metal ion binding, we computed the average buried area and p*K*_a_ of Tyr residues along a molecular dynamics simulation of the SY7 fibril.^40^ The average buried area is 25%, and the average p*K*_a_ is 11.5, confirming that Tyr residues are mostly exposed and available for binding and that further stabilization to the metal–fibril interaction could come from a fraction of deprotonated Tyr side chains.

Overall, computational calculations confirm and explain how Tyr-rich fibrils can coordinate to metallic ions.

### 4 Characterization of Prion-Inspired Fibrils Bound to Metallic Cations by Electron Microscopy

Having established that the fibrils can retain Cu^2+^, Ni^2+^, Co^2+^, and Zn^2+^, we investigated the impact of metal cations binding on their morphology. First, NY7, QY7, SY7, and GY7 fibrils were examined by transmission electron microscopy (TEM) prior to their incubation with metals, showing well-defined fibrils (Figure S5A) of some micrometers in length, and a width of 8 to 20 nm, consistent with our previous data.^41^ Additionally, Thioflavin-T (Th-T) binding confirmed the amyloid nature of these assemblies (Figure S5B).

**Figure 3:**
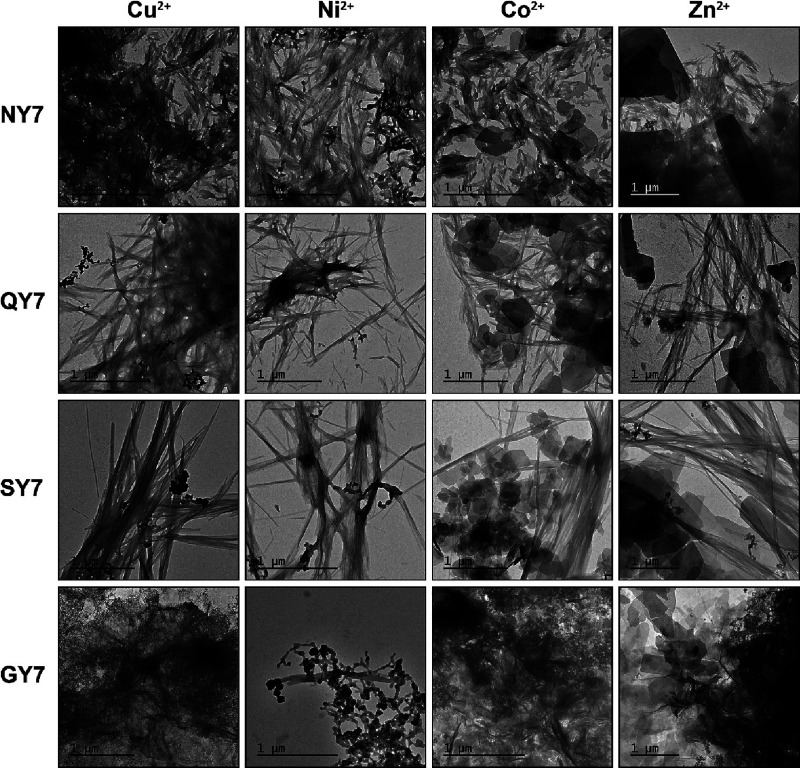
TEM micrographs of fibrils incubated with Cu^2**+**^, Ni^2+^, Co^2+^, and Zn^2+^. Representative transmission electron micrographs of the deposited fibrils onto TEM grids. NY7, QY7, SY7, and GY7 self-assembled fibrils were incubated overnight at 25 °C in the presence of 200 mM CuCl_2_, NiSO_4_, CoCl_2_, and ZnCl_2_ under soft agitation. All samples were deposited onto carbon-coated copper grids except Cu^2+^ samples, which were deposited onto carbon-coated gold grids. Scale bars correspond to 1 μm.

Next, NY7, QY7, SY7, and GY7 fibrils incubated with Cu^2+^ were deposited on carbon-coated gold grids, while NY7, QY7, SY7, and GY7 fibrils incubated with Ni^2+^, Co^2+^, and Zn^2+^ were deposited on carbon-coated copper grids and visualized by TEM. The presence of fibrils resembling in width those in the absence of metals was visible in all cases (Figure 3). Notably, the binding of metals to the fibrils allowed their visualization without the need of using uranyl acetate as contrasting agent, as it was needed in the case of nonincubated fibrils. In images of fibrils incubated with Cu^2+^ and Ni^2+^ electrodense dots over the fibrils were evidenced, whereas in images of Co^2+^- and Zn^2+^-incubated fibrils, the presence of polygonal depositions was observed. This suggests that not only do the fibrils bind the metals, but they also facilitate their self-assembly into macromolecular structures.

We selected the NY7 and QY7 peptides to demonstrate that the metalized fibrils bind Th-T (Figure S6) and display a predominant β-sheet structure, as measured by Fourier transform infrared (FTIR) spectroscopy in the amide I region of the spectrum (Figures S7 and S8).

To provide further confirmation of the divalent metal cation attachment in the deposited fibrils, electronic diffraction (ED) and energy dispersive X-ray diffraction (EDX) techniques were employed. First, the distances of the different diffraction patterns were measured (Tables S2 to S5) from the ED micrographs (Figure S9) and compared to the reference crystalline diffraction pattern for each element available in the CaRIne Crystallography Software (France) database. All diffraction patterns of metallized fibrils matched the reference pattern for the respective metal. EDX spectra revealed an enrichment in the incubated divalent cation element for all fibrillar samples: Cu^2+^ (Figure S10); Ni^2+^ (Figure S11); Co^2+^ (Figure S12); and Zn^2+^ (Figure S13).

Altogether, the results in this section provided solid evidence for the presence and identity of metal divalent cations coordinated to NY7, QY7, SY7, and GY7 fibrils.

### 5 Esterase Activity of Prion-Inspired Fibrils Decorated with Cu^2+^ and Zn^2+^

Hydrolytic enzymes split different groups of biomolecules such as esters, peptides, and glycosides, in many cases thanks to the coordination of a metal ion at the catalytic center. Although His is the preferred metal coordinating residue, Tyr can also participate in the binding in some metalloproteins.^36−39^ His–metal coordination has been exploited for the design of artificial nanozymes.^5,10,26,31^ We assayed if our peptides may act as a hydrolytic enzyme-like scaffold when bound to Cu^2+^ and Zn^2+^ and thus if we could generate an artificial Tyr-rich hydrolytic amyloid for the first time.

Esterase activity is typically assayed using *p*-nitrophenyl acetate (pNPA) as the substrate and monitoring the appearance of the yellow-colored product *p*-nitrophenol (pNP). Therefore, we used pNPA to assess the esterase activity of fibrils when coordinated with Cu^2+^ and Zn^2+^. Kinetic analysis revealed that all metal-coordinated heptapeptide fibrils exhibited esterase activity (Figure S14), acting thus as hydrolase-like catalysts. Remarkably, the activity of the soluble l-Tyr amino acid in the presence of metal cations is low (Cu^2+^) or negligible (Zn^2+^), implying that it is the three-dimensional structure of the fibril that provides an appropriate microenvironment for the metal-dependent catalytic activity.

**Figure 4:**
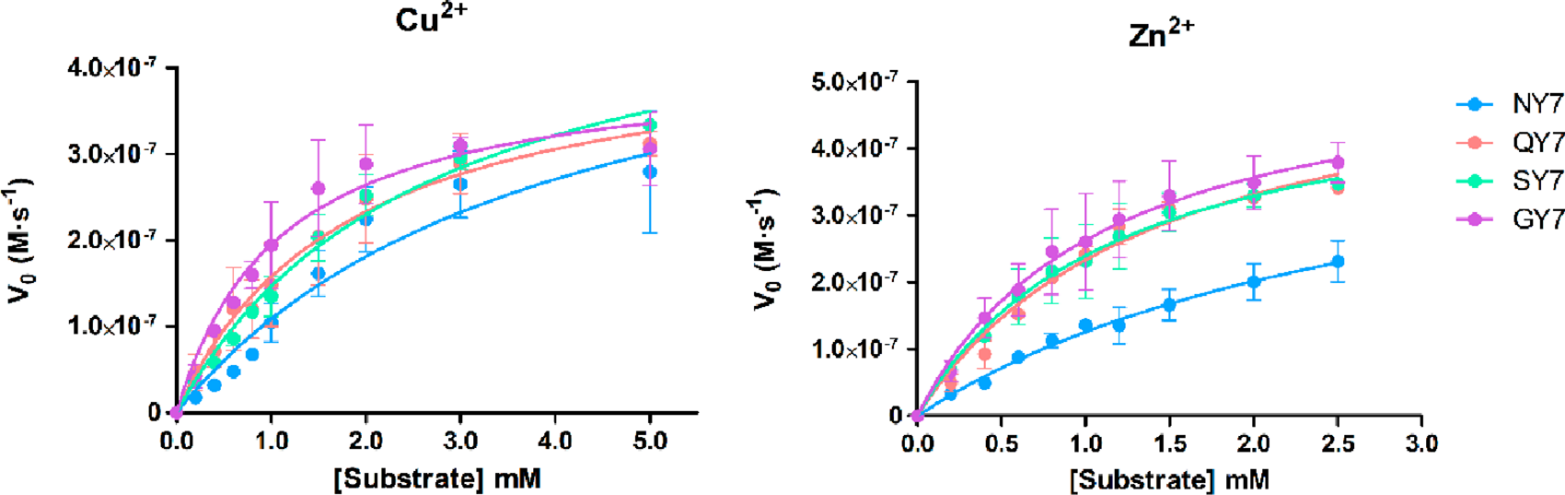
Kinetic curve fitting to the Michaelis–Menten equation for heptapeptide fibril esterase activity in the presence of Cu^2**+**^ and Zn^2**+**^. Esterase activity was detected by measuring the increasing absorbance at 405 nm along the time corresponding to the appearance of pNP yellow-colored product in 25 mM HEPES pH 8.0 with 1 mM CuCl_2_ and 25 mM Tris-HCl pH 8.0 with 1 mM ZnCl_2_, respectively. Obtained data were fitted to the Michaelis–Menten equation with GraphPad PRISM 5.0 software for Cu^2+^ (left graph) and Zn^2+^ (right graph). Data correspond to the mean of three independent experiments (± standard deviation).

Kinetic parameters of the esterase activity in the presence of metals were obtained by fitting the reactions in a substrate concentration range from 0.2 to 2.5 (Zn^2+^) or 0.2 to 5 mM (Cu^2+^) to the Michaelis–Menten equation (Figure 4). The Michaelis constants (*K*_M_) and maximal velocity of the reactions (*K*_cat_) were calculated (Table S6).

The four heptapeptides in the presence of Cu^2+^ presented a *K*_M_ ranging between 1.4 and 3.8 mM and a *k*_cat_ around 0.006 s^–1^. Subtle differences in the catalytic efficiency between the peptides were observed, with GY7 corresponding to the most efficient fibril with a *k*_cat_/*K*_M_ = 3.8 ± 1.4 M^–1^·s^–1^. Similarly, the Zn^2+^-containing heptapeptide fibrils displayed *K*_M_ in the mM range, between 1.1 and 3.0 mM, and *k*_cat_ around 0.006 s^–1^. GY7 exhibited the highest catalytic efficiency, with a *k*_cat_/*K*_M_ = 6.0 ± 2.3 M^–1^·s^–1^.

Hydrolysis of pNPA by Zn^2+^-bound prion-inspired fibrils and, in particular, by the modeled SY7 scaffold (*k*_cat_/*K*_M_ = 5.8 ± 2.07 M^–1^·s^–1^) is less efficient than that reported by Korendovych and co-workers for the more hydrophobic, His-based IHIHIQI heptapeptide (IH7) (*k*_cat_/*K*_M_ = 62 ± 2 M^–1^·s^–1^).^24^ Comparison of B3LYP-D3 calculations for model systems representing the metal site in IH7 [Zn^2+^(His)_3_OH^–^]^+^ and SY7 [Zn^2+^(Tyr)_3_OH^–^]^+^ showed that the energy barrier for the OH^–^ attack is considerably lower in IH7 (9 kcal·mol^–1^) than in SY7 (19 kcal·mol^–1^). This is mainly because the Zn–OH^–^ bond is weaker in [Zn^2+^(His)_3_OH^–^]^+^ than in [Zn^2+^(Tyr)_3_OH^–^]^+^. Furthermore, deprotonation of coordinated water is more favorable in IH7 due to the presence of a fourth His able to accept the proton.

Despite, as expected, His-based fibrils performing better than Tyr-based ones, our data show that all fibrils, when coordinated with Cu^2+^ and Zn^2+^, behave as esterases, with their kinetic parameters depending somehow on the nature of differential polar side chains in each sequence, since the Tyr content and disposition are common to all of them.

### 6 Carbonic Anhydrase Activity of Prion-Inspired Fibrils Decorated with Cu^2+^ and Zn^2+^

Carbonic anhydrases are among the most ubiquitous enzymes since they are involved in multiple physiological functions such as pH homeostasis, calcification, bone resorption, and photosynthesis. Moreover, they display a pivotal role in the transport of carbon dioxide, by catalyzing the reversible hydration of CO_2_ into bicarbonate (HCO_3_^–^) and a proton (H^+^), its activity being strictly dependent on the coordination of Zn^2+^ at the catalytic center.

The search for artificial CA mimics has concentrated considerable efforts.^8,15^ However, to the best of our knowledge, a Tyr-based amyloid catalytic system has not yet been yet explored. Accordingly, we aimed to investigate if our fibrils could catalyze the hydration of carbon dioxide and water in the presence of Zn^2+^ and Cu^2+^, producing H^+^ and an acidification of the solution.

**Figure 5:**
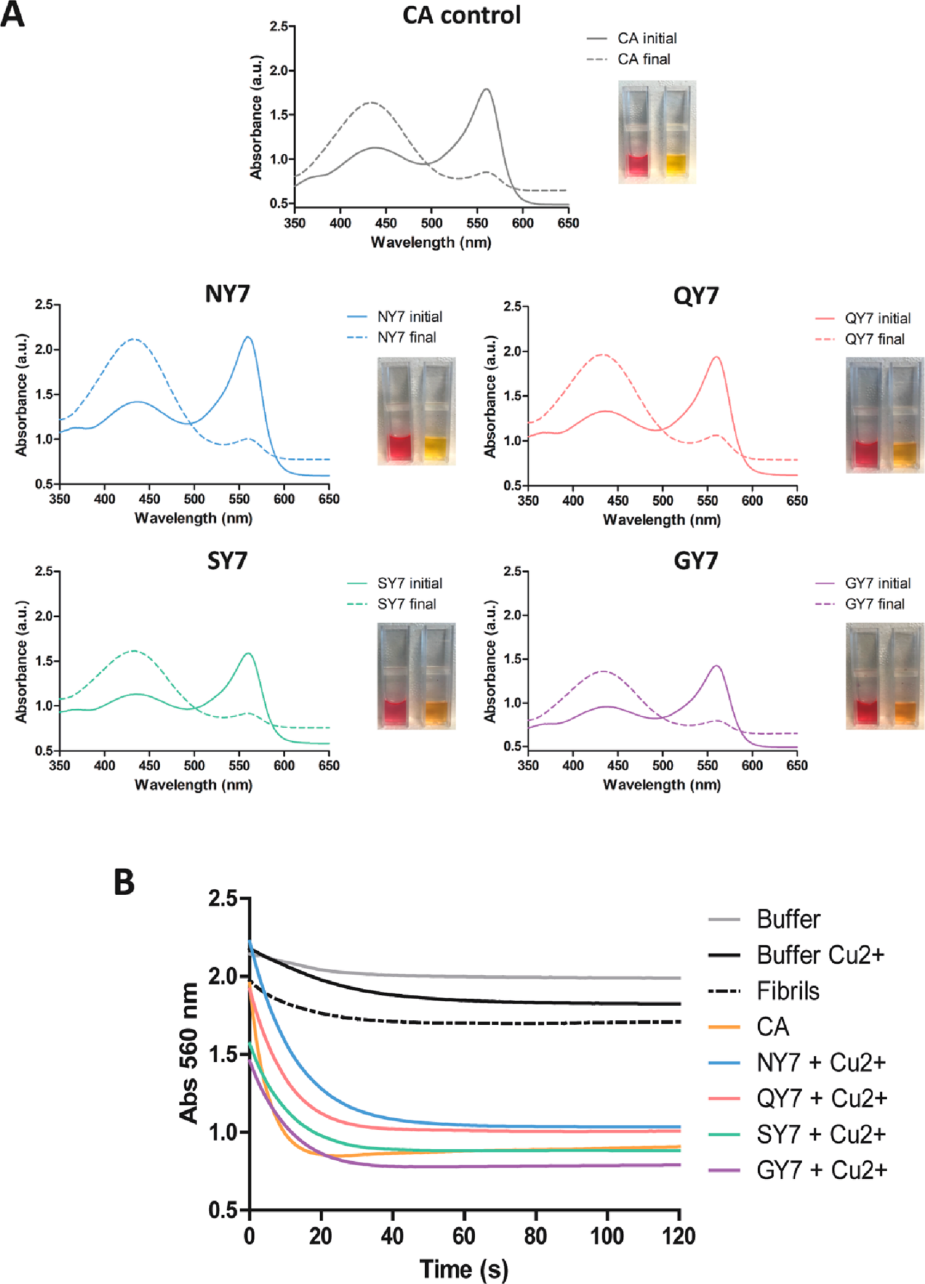
Carbonic anhydrase activity of the fibrils in the presence of Cu^2+^. (A) Carbonic anhydrase (CA) activity was detected by measuring absorbance of the phenol red pH indicator in the presence of NY7 (blue), QY7 (red), SY7 (green), and GY7 (purple) and positive control with CA at 50 nM (gray). Initial (continuous line) and final (discontinuous line) spectra were acquired between 350 and 650 nm. The image shows the phenol red coloration corresponding to the initial (red) and final (yellow) samples. (B) Time-dependent decrease in absorbance at 560 nm of phenol red incubated with CO_2_-treated deionized water. Fibril colors as in (A), negative controls of buffer alone (gray), buffer with Cu^2+^ (black), QY7 fibrils without metal (black discontinuous), and CA at 50 nM (orange) are indicated.

**Figure 6:**
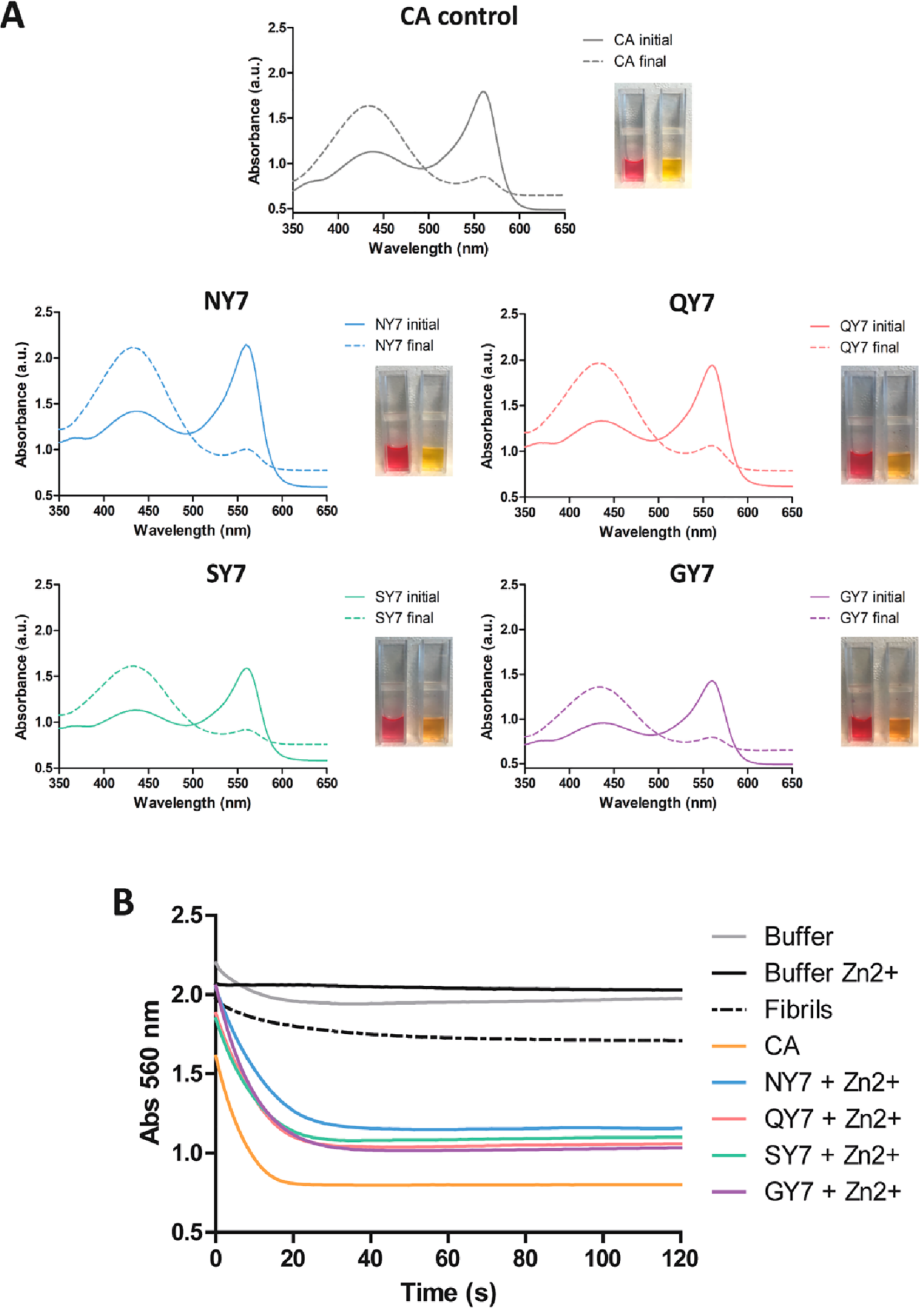
Carbonic anhydrase activity of the fibrils in the presence of Zn^2+^. (A) Carbonic anhydrase (CA) activity was detected by measuring absorbance of the phenol red pH indicator in the presence of NY7 (blue), QY7 (red), SY7 (green), and GY7 (purple) and positive control with CA at 50 nM (gray). Initial (continuous line) and final (discontinuous line) spectra were acquired at between 350 and 650 nm. The image shows the phenol red coloration corresponding to the initial (red) and final (yellow) samples. (B) Time-dependent decrease in absorbance at 560 nm of phenol red incubated with CO_2_-treated deionized water. Fibril colors as in (A), negative controls of buffer alone (gray), buffer with Zn^2+^ (black), QY7 fibrils without metal (black discontinuous), and CA at 50 nM (orange) are indicated.

The CA activity of the fibrils was followed using phenol red, which presents red coloration at alkaline pH and changes to yellow at acidic pH. Absorbance spectra of self-assembled heptapeptides in the range of 350–650 nm were acquired before and 2 min after the addition of carbonated H_2_O. As shown in Figure 5A and Figure 6A, the initial spectra exhibited a peak at 560 nm and a second lower peak at 430 nm, characteristic of an alkali medium with a pH around 8.0, whereas after incubation it displayed a significant decrease in the 560 nm peak and a maximum at 430 nm, indicating a drop in the pH due to the generation of H^+^. In this experiment, commercial CA protein was used as a positive control of the reaction.

Next, CA kinetics were followed by measuring the decrease in absorbance at 560 nm along time for 2 min (Figures 5B and 6B), and the relative activity was calculated (Table S7). Negative controls in the absence of heptapeptide fibrils, with or without the metal ions, and fibrils without added metals exhibited residual activity. As one would expect, wild-type CA protein, used in the experiment as a positive control, promoted a high and fast reduction in absorbance. Importantly, all Cu^2+^ and Zn^2+^ incubated fibrils were active, displaying significant CA activity, in some cases resembling that of the natural enzyme in the same conditions (Figures 5B and 6B and Table S7).

Overall, our results demonstrate that the metallized prion-inspired fibrils are endowed with hydrolytic catalytic activity, allowing fixation and hydration of CO_2_.

## Conclusions

In this study, we have investigated the coordination of Cu^2+^, Co^2+^, Ni^2+^, and Zn^2+^ ions by NY7, QY7, SY7, and GY7 amyloid fibrils. Unlike catalytic amyloids reported to date, the short peptides forming these fibrils lack the characteristic His residues but are instead enriched with Tyr residues. Given that the phenolate oxygen in Tyr has been reported to coordinate divalent metal cations in certain metalloproteins and provided that a large number of phenolic hydroxyl groups are exposed to solvent at the surface of our prion-inspired fibrils, we hypothesized that these scaffolds could coordinate metal ions and thus become metallized.

Spectrophotometric and ICP-OES measurements consistently demonstrate that prion-inspired fibrils possess remarkable metal retention capacity. Docking simulations and electronic structure calculations indicate that up to three Tyr residues can simultaneously coordinate to either Cu^2+^ or Zn^2+^ through their phenolic rings, with a water molecule occupying the vacant site. Specifically, the coordination of Cu^2+^ involves Tyr in two or three strands, whereas Zn^2+^ coordinates with Tyr in two different strands. Quantum chemical calculations indicate that oxygen (Tyr) bonding distances are similar to those observed in metalloproteins, lending support to the stable interactions between fibrils and metallic ions observed experimentally.

TEM, electronic diffraction, and energy dispersive X-ray diffraction spectroscopy techniques provided compelling evidence for the presence and identity of metallic divalent cations coordinated to NY7, QY7 SY7, and GY7 fibrils. The images suggested that the fibrils not only bind to metals but also may facilitate polymerization on the fibril surface into electrodense spherical or polygonal structures, a phenomenon that likely contributes to the high metal/peptide ratio observed by ICP-OES. This metal ion sequestration capability suggests that the fibrils may be useful for environmental remediation, acting as metallic traps for purifying contaminated water and soil. Importantly, unlike previous amyloid scavengers, whose formation requires high temperatures and acidic conditions, our prion-inspired scaffolds self-assemble into highly stable amyloids under mild conditions, making them environmentally friendly.

The most remarkable feature of the prion-inspired amyloids is their capacity to function as metalloenzymes when they are coordinated to metal ions. Unlike most natural enzymes and designed catalytic supramolecular assemblies, containing a His as the nucleophile, our amyloids coordinate Zn^2+^ and Cu^2+^ via the aromatic Tyr residues that face the solvent in the β-strand ladder. Kinetic analysis of the esterase activity reveals that the heptapeptide fibrils exhibit substrate-concentration-dependent Michaelis–Menten kinetics, thus functioning as true metalloenzymes, despite the absence of a His triad. Notably, the esterase activity of a soluble l-Tyr amino acid in the presence of metal cations is low, indicating that it is the three-dimensional structural framework of the fibril that provides an appropriate microenvironment for the metal-dependent catalytic activity, consistent with computational calculations.

The esterase catalytic efficiency of our Tyr-based fibrils is lower than that of His-based amyloids, which is consistent with the evolutionary selection of His rather than Tyr at the active site of current hydrolases. In any case, our findings support the notion that amyloid-like structures formed by short and low-complexity peptides could act as catalysts at the origin of life while expanding the amino acid sequence and composition space compatible with the expression of hydrolytic activities in these ancestral polymers.

Importantly, in many of the reported His-based catalytic amyloids, the presence of the metal cation during the assembly reaction is required for amyloid formation, since it provides contacts necessary for interstrand interactions. In contrast, the peptides described herein self-assemble spontaneously in the absence of a metallic cofactor but can bind it in their mature fibrillar form. This property enables them to function as both metal scavengers and nanoenzymes and may have implications for their activity in an ancient world context. This allows the uncoupling of the site of fibril synthesis from the site where they can express their activity; thus, their action may be compartmentalized in microenvironments with appropriate metal ion concentrations.

Finally, we have successfully developed for the first time a minimalistic artificial amyloid-like carbonic anhydrase that utilizes Tyr–metal coordination for its catalytic activity. These fibrillar nanoenzymes exhibit a remarkable capacity to capture CO_2_, opening an avenue for their utilization in a wide range of fields, including biotechnology, environmental science, and biomedicine, where their biodegradable and innocuous nature could prove to be advantageous.

## Methods

### Peptide Preparation

Synthetic heptapeptides NY7 (Ac-NYNYNYN-NH_2_), QY7 (Ac-QYQYQYQ-NH_2_), SY7 (Ac-SYSYSYS-NH_2_), and GY7 (Ac-GYGYGYG-NH_2_) were purchased from CASLO ApS (Scion Denmark Technical University). Lyophilized peptides were dissolved in 1,1,1,3,3,3-hexafluoro-2-propanol to obtain a 10 mM stock solution, aliquoted, and frozen at −80 °C.

### Peptide Aggregation Reactions

NY7, QY7, and SY7 peptides were diluted from a 10 mM stock to a final concentration of 250 μM in 100 mM potassium phosphate, pH 7.0. Solutions were incubated in quiescent conditions for 7 days at 25 °C. We have demonstrated computationally and experimentally^40,41^ that the GY7 peptide displays a higher critical concentration for fibrillation; therefore, it was diluted to a final concentration of 500 μM in 100 mM potassium phosphate, pH 7.0.

### Transmission Electron Microscopy

For the preparation of TEM samples, 10 μL of NY7, QY7, SY7, and GY7 fibrils incubated with Ni^2+^, Co^2+^, and Zn^2+^ were deposited onto carbon-coated copper grids, and NY7, QY7, SY7, and GY7 fibrils incubated with Cu^2+^ were deposited onto carbon-coated gold grids for 10 min. Liquid excess was removed with filter paper. No negative staining was performed. As controls, NY7, QY7, SY7, and GY7 fibrils without incubation with divalent metal cations were deposited onto carbon-coated copper grids for 10 min, and negative staining was performed with 2% w/v uranyl acetate solution for 1 min. Grids were scanned using a JEM 1400 transmission electron microscope (JEOL Ltd., Japan) operating at 80 kV, and micrographs were acquired with a CCD GATAN ES1000W Erlangshen camera (Gatan Inc., USA).

### Thioflavin-T Binding

Thioflavin-T (Th-T) dye binding to non-metallized NY7, QY7, SY7, and GY7 fibrils or metallized NY7 and QY7 fibrils was analyzed to confirm the amyloid character. Self-assembled peptides were diluted 1:10 in 100 mM potassium phosphate, pH 7.0, with 25 μM Th-T. The Th-T fluorescence signal was measured on a Jasco FP-8200 fluorescence spectrophotometer (Jasco Corporation, Japan) in the 460–600 nm range, using an excitation wavelength of 445 nm and with an excitation and emission bandwidth of 5 nm.

### Divalent Metal Cation Binding to Self-Assembled Heptapeptide Fibrils

NY7, QY7, SY7, and GY7 self-assembled fibrils were centrifuged at 12000*g* for 30 min and resuspended in Milli-Q water in half the initial volume. Divalent metal cations Cu^2+^, Ni^2+^, Co^2+^, and Zn^2+^ stock solutions were prepared at 500 mM using CuCl_2_, NiSO_4_, CoCl_2_, and ZnCl_2_ salts, respectively (Sigma-Aldrich, USA). Centrifuged NY7, QY7, SY7, and GY7 fibrils were incubated in the presence of increasing amounts of each divalent cation: Cu^2+^ was added at 5, 10, 50, 100, and 200 mM; Ni^2+^ was added at 50, 100, and 200 mM; Co^2+^ was added at 20, 50, 100, and 200 mM; Zn^2+^ was added at 5, 10, 20, and 50 mM. All the reactions were incubated at RT with soft agitation. Samples were centrifuged at 12000*g* for 30 min, and pellet and supernatant were separated and preserved for further analysis.

### Quantification of NY7, QY7, SY7, and GY7 Peptides

Heptapeptide fibrils from day 7 were quantified from the soluble fraction after centrifugation of the reaction. Then the supernatant was diluted 1:10 and incubated with 2.5 M guanidinium thiocyanate (GITC) in 100 mM potassium phosphate buffer for a minimum of 12 h under soft agitation to reach the equilibrium. Calibration curves using a known concentration of QY7, NY7, SY7, and GY7 peptides ranging from 0 to 50 μM were incubated under the same conditions. Tyrosine intrinsic fluorescence was acquired on a Jasco FP-8200 fluorescence spectrophotometer (Jasco Corporation, Japan) in the range 280–400 nm, exciting at 268 nm wavelength and using an excitation and emission bandwidth of 5 nm at 25 °C. Fluorescence emission at 303 nm, which corresponds to the tyrosine emission maximum, was used for quantification, using the equation from the corresponding calibration curve.

### Spectrophotometric Quantification of Divalent Metal Cations

Metallized NY7, QY7, SY7, and GY7 fibrils were obtained as previously indicated, and their supernatants were preserved for spectrophotometric analysis. Calibration curves for Cu^2+^, Ni^2+^, and Co^2+^ were obtained measuring absorbance at 235 nm for Cu^2+^-incubated samples, at 393 nm for Ni^2+^-incubated samples, and at 512 nm for Co^2+^-incubated samples, using increasing concentrations of each divalent cation (0 to 200 mM for Cu^2+^, 0 to 500 mM for Ni^2+^, 0 to 200 mM for Co^2+^). Molar extinction coefficients (ε) were obtained experimentally by measuring the absorbance of stock divalent cation solution at different concentrations and calculated from the slope of the obtained equations: Cu^2+^_ε235 nm_ = 189 M^–1^·cm^–1^; Ni^2+^_ε393 nm_ = 4.8 M^–1^·cm^–1^; Co^2+^_ε512 nm_ = 6.0 M^–1^·cm^–1^. For the Zn^2+^ spectrophotometric quantification, Zincon dye was used since the Zn^2+^ absorbance maximum is under 200 nm. Zincon dye detects Zn^2+^ in solution, showing a color change from red to blue with an absorbance maximum at 620 nm in the presence of Zn. A Zincon monosodium salt (Sigma-Aldrich, USA) stock solution was prepared at 2 mM in 20 mM NaOH. The calibration curve for Zn^2+^ was obtained by incubating increasing Zn^2+^ concentrations (0 to 80 μM) with 100 μM Zincon and measuring absorbance at 620 nm. The molar extinction coefficient for the Zn–Zincon complex was experimentally calculated from the slope of the obtained equation: Zn–Zincon_ε620 nm_ = 22 800 M^–1^·cm^–1^. All of the measurements were performed in a Specord200 Plus spectrophotometer (Analytik Jena, Germany). Data were analyzed calculating the retention percentage for each peptide and cation, tacking as a reference each concentration point used to incubate the fibrils, obtaining the final percentage of the cation retained by fibrils.

### Fourier Transform Infrared Spectroscopy

NY7 and QY7 fibrils were centrifuged at 12000*g* for 30 min, resuspended in Milli-Q water, and incubated with divalent metal cations Cu^2+^, Co^2+^, and Zn^2**+**^ at a final concentration of 200 mM, overnight at RT. Samples were washed twice with water, and fibrils were recovered for FTIR analysis.

Infrared spectra were collected using a Bruker Platinum Alpha Eco-ATR FTIR spectrometer (Bruker Optics, USA). Fibers were deposited on the diamond ATR crystal, and solvent was evaporated in the stream of nitrogen. FTIR spectra were recorded between 1700 and 1600 cm^–1^ in 32 acquisitions at a resolution of 1 cm^–1^. Spectra were corrected for background absorption and normalized.

### Electronic Diffraction

Grids prepared for TEM visualization were also used for electronic diffraction in a JEM-2011 transmission electron microscope (JEOL Ltd., Japan) operating at 200 kV. Characteristic distances of diffraction patterns of Cu^2+^, Ni^2+^, Co^2+^, and Zn^2+^ were measured from the obtained micrographs using ImageJ software (NIH, USA).

### Energy Dispersive X-ray Spectroscopy

Grids prepared for TEM visualization were used for EDX spectroscopy analysis. The EDX spectrum for each sample was acquired in a JEM-2011 transmission electron microscope (JEOL Ltd., Japan) operating at 200 kV, equipped with an EDS X-max detector (Oxford Instruments, UK). Spectra were analyzed using INCA Software (ETAS group, Germany) and represented with GraphPad Prism 5.0 (GraphPad Software, USA).

### Inductively Coupled Plasma Atomic Optic Emission Spectrometry

NY7, QY7, SY7, and GY7 assembled fibrils were incubated in the presence of 200 mM of each divalent cation in a 500 μL final volume as previously described, corresponding to a saturation cation concentration. A 500 μL amount of nonincubated samples with divalent metal cations was used as controls. Incubated samples were centrifuged at 12000*g* for 30 min, and pellets were stored for their further analysis. Pelleted fibrils were resuspended in an HNO_3_ 65% solution (Merck Suprapur, Germany) and heated in a DINKO D-65 heating block to promote peptide digestion. Digestion products were injected in an inductively coupled plasma optic emission Optima 4300DV mass spectrometer (PerkinElmer, USA) to quantify divalent metal cation content.

### Esterase Activity

NY7, QY7, SY7, and GY7 assembled fibrils were prepared as described and isolated by centrifugation at 12000*g* for 30 min. Esterase activity was tested in the presence of Cu^2+^ and Zn^2+^, requiring different buffering conditions: 25 mM HEPES pH 8.0 for Cu^2+^ and 25 mM Tris HCl pH 8.0 for Zn^2+^. Samples were incubated in the presence of 1 mM CuCl_2_ and 1 mM ZnCl_2_ at 25 °C in their respective buffers. Peptide concentration used in all assays corresponds to 100 μM.

The reactions were performed in 96-well plates and measured every 10 min until a plateau was reached in a Victor3 plate reader (PerkinElmer, USA), acquiring absorbance at 405 nm. *p*-Nitrophenyl acetate (*p*-NPA) and *p*-nitrophenol (*p*-NP) (Sigma-Aldrich, Germany) stock solutions were freshly prepared at 0.1 M in acetonitrile and diluted to the desired final concentrations. Extinction molar coefficients for *p*-NP in each buffering condition were experimentally obtained from the slope of calibration curves, which were obtained from three independent replicas and measured in duplicate. Obtained values were as follows: for Cu^2+^ experiments ε_405 nm_ = 11 670 M^–1^·cm^–1^ and for Zn^2+^ experiments ε_405 nm_ = 13 409 M^–1^·cm^–1^.

Preliminary kinetic measurements were performed in the presence of 450 μM *p*-NPA and 100 μM fibrils previously incubated with the corresponding divalent metallic cation (Cu^2+^ or Zn^2+^). Corresponding buffer signal was subtracted from all the samples. A 50 μM concentration of l-Tyr was measured as a control. Data were analyzed using GraphPad Prism Software and fitted to a hyperbolic equation.

Kinetic parameter calculations were further obtained for both conditions at a final peptide concentration of 100 μM. For Cu^2+^-incubated peptides, the substrate (*p*-NPA) concentration used ranged from 0.2 to 5 mM, and for the Zn^2+^ substrate (*p-*NPA) the concentration ranged from 0.2 to 2.5 mM. Reported results correspond to the average of at least three independent measurements. Kinetic parameters were obtained by fitting the data to the Michaelis–Menten equation [*V*_0_ = *k*_cat_·[E]_0_·[S]_0_/(*K*_M_ + [S]_0_)] using GraphPad Prism 5.0 software.

### Carbonic Anhydrase Activity Assay

NY7, QY7, SY7, and GY7 assembled fibrils were prepared as described and isolated by centrifugation at 12000*g* for 30 min. Carbonic anhydrase was tested in the presence of Cu^2+^ and Zn^2+^, requiring different buffering conditions: 25 mM HEPES pH 8.0 for Cu^2+^ and 25 mM Tris HCl pH 8.0 for Zn^2+^. Samples were incubated in the presence of 1 mM CuCl_2_ and 1 mM ZnCl_2_ overnight at 25 °C in their respective buffers under soft agitation. Peptide concentration used in all assays corresponds to 100 μM. Phenol red pH indicator was used to follow the pH modification of the reaction at a final concentration of 100 μM in the corresponding buffers.

Carbon dioxide (CO_2_)-saturated water was freshly prepared by bubbling CO_2_ gas flow into 100 mL of Milli-Q water in a round-bottom crystal flask with a flow rate at 5 psi under ice-cold conditions for 1 h. A 150 μL amount of 100 μM phenol red in the corresponding buffers was used to perform the experiments in the presence and in the absence of self-assembled peptides. A 50 μL amount of CO_2_-saturated water was added to the samples. Reactions were performed in a Specord200 Plus spectrophotometer (Analytik Jena, Germany) measuring the change in the absorbance at 560 nm. Additionally, initial and final spectra were acquired in the 350–650 nm range. Carbonic anhydrase at 50 nM was used as a positive control.

### Computational Details

Docking calculations were carried out on our previously built 40-strand fibril model,^40^ with the GOLD software package,^45^ applying the improvement designed for metal ion docking^46^ and using the GoldScore (GS) scoring function, a robust scoring for posing predictor. Genetic algorithm (GA) parameters were set to 50 GA runs and a minimum of 100 000 operations. The rest of the parameters were set to default. Protein residues were considered rigid, except the tyrosine ones included in the evaluation sphere of 7 Å centered on the binding site.

Once the possible binding sites were identified, we cut out from the fiber those strands directly involved in the metal coordination and ran quantum chemical calculations with the hybrid B3LYP density functional method^47,48^ and applying the Grimme’s correction for dispersion (D3).^49^ The scalar relativistic Stuttgart–Dresden SDD pseudopotential and its associated basis set were used for copper and zinc, and the standard 6-31G(d) basis set was used for the rest of the atoms. Optimizations were done fixing the position of the terminal C_a_ of each strand to simulate the restraints imposed by the fiber. Solvent effects were accounted for with the SMD implicit solvation model.^50^ For NPA hydrolysis simulations we used the larger 6-31+G(d,p) basis set and the CPCM implicit solvation model. All calculations were performed using the GAUSSIAN16 software package.^51^

## Supporting Information Available

Table containing divalent cation quantification by ICP-OES; tables containing electronic diffraction distances from Cu^2+^-incubated fibrils, Ni^2+^-incubated fibrils, Co^2+^-incubated fibrils, and Zn^2+^-incubated fibrils; table containing kinetic parameters of the fibrils’ esterase activity in the presence of Cu^2+^; table containing kinetic parameters of the carbonic anhydrase activity of the self-assembled fibrils in the presence of Cu^2+^ and Zn^2+^; models of GY7 fibrils; experimental determination of molar extinction coefficient for divalent cations Ni^2+^, Cu^2+^, Co^2+^, and Zn^2+^ (with Zincon complex); graphical representation of the fibrils’ retention capacity for divalent cations Ni^2+^, Cu^2+^, Co^2+^, and Zn^2+^; optimized structures for Cu^2+^ and Zn^2+^ interacting with the p system of tyrosines; biophysical characterization of NY7, QY7, SY7, and GY7 fibrils; fluorescence emission spectra of Th-T in the presence of NY7 and QY7 fibrils incubated with Cu^2+^, Zn^2+^, and Co^2+^; FT-IR absorbance spectrum in the amide I region of NY7 fibrils alone or incubated with Cu^2+^, Zn^2+^, and Co^2+^; FT-IR absorbance spectrum in the amide I region of QY7 fibrils alone or incubated with Cu^2+^, Zn^2+^, and Co^2+^; electronic diffraction pattern micrographs from heptapeptide fibrils incubated with Ni^2+^, Cu^2+^, Co^2+^, and Zn^2+^; electronic dispersive X-ray spectroscopy spectra from heptapeptides incubated with Cu^2+^, Ni^2+^, Co^2+^, and Zn^2+^; fibrils’ esterase activity kinetics in the presence of Cu^2+^ and Zn^2+^ (PDF)

## Notes

A previous draft of this work was partially published as a preprint: Diaz-Caballero, M. Prion inspired assemblies to build up functional bionanomaterials. 2021. Universitat Autònoma de Barcelona. Departament de Bioquímica i Biologia Molecular. Dipòsit Digital de Dades de la UAB (https://ddd.uab.cat/record/238200, accessed Aug 21, 2023).
