# Supplementary material for: Amyloid Fibrils Formed by Short Prion-Inspired Peptides Are Metalloenzymes
Source: ACS Nano. 2023 Aug 30;17(17):16968–79. doi: 10.1021/acsnano.3c04164 (PMC10510724; doi:10.1021/acsnano.3c04164)
Supplement: Supplementary file 1 — nn3c04164_si_001.pdf [file nn3c04164_si_001.pdf]

Supplementary Material for:

Amyloid Fibrils Formed by Short Prion-Inspired Peptides Are Metalloenzymes

*Susanna Navarro[1], Marta Díaz-Caballero[1], Francesca Peccati,[2] Lorena Roldan-Martín[3] Mariona Sodupe[3] and Salvador Ventura[1]**

[1] Institut de Biotecnologia i de Biomedicina (IBB) and Departament de Bioquímica i Biologia Molecular; Universitat Autònoma de Barcelona; 08193 Bellaterra (Barcelona), Spain.

[2] Basque Research and Technology Alliance (BRTA), Center for Cooperative Research in Biosciences (CIC bioGUNE), 48160 Derio, Spain

[3] Departament de Química, Universitat Autònoma de Barcelona, 08193 Bellaterra (Barcelona), Spain.

Table S1. Divalent cations quantification by Inductively Coupled Plasma Atomic Optical Emission Spectrometry (ICP-OES). ICP-OES was used to calculate the quantity of divalent cations Cu^{2+}, Ni^{2+}, Co^{2+} and Zn^{2+} retained by the fibrils in 500 µL final volume. The values correspond to the mean of duplicates from 2 independent experiments (± standard deviation).

Cation	Peptide	µg cation			
		Cu^{2+}	Ni^{2+}	Co^{2+}	Zn^{2+}
Cu^{2+}	NY7	206 ± 51	< 0.16	< 0.16	< 0.16
	QY7	330 ± 59	< 0.16	< 0.16	< 0.16
	SY7	329 ± 30	< 0.16	< 0.16	< 0.16
	GY7	384 ± 17	< 0.16	< 0.16	< 0.16
Ni^{2+}	NY7	< 0.16	96 ± 1	< 0.16	< 0.16
	QY7	< 0.16	175 ± 6	< 0.16	< 0.16
	SY7	< 0.16	238 ± 57	< 0.16	< 0.16
	GY7	< 0.16	100 ± 72	< 0.16	< 0.16
Co^{2+}	NY7	< 0.16	0.42	231 ± 28	< 0.16
	QY7	< 0.16	0.55	325 ± 6	< 0.16
	SY7	< 0.16	0.585	356 ± 35	< 0.16
	GY7	0.22	0.39	250 ± 17	< 0.16
Zn^{2+}	NY7	< 0.16	< 0.16	< 0.16	195 ± 25
	QY7	< 0.16	< 0.16	< 0.16	298± 47
	SY7	< 0.16	< 0.16	< 0.16	271± 77
	GY7	< 0.16	< 0.16	< 0.16	216 ± 59
Control	NY7	< 0.16	< 0.16	< 0.16	< 0.16
	QY7	< 0.16	< 0.16	< 0.16	< 0.16
	SY7	< 0.16	< 0.16	< 0.16	< 0.16
	GY7	< 0.16	< 0.16	< 0.16	< 0.16

Table S2. Electronic diffraction distances from Cu^{2+} incubated fibrils. Distances from Cu^{2+} electronic diffraction pattern for each sample were obtained from electronic diffraction micrographs shown in Figure S6, obtained by TEM, using ImageJ Software (NIH, USA). [a] Reference electron diffraction pattern distances from Cu^{2+} are shown in blue and were obtained from the database of CaRIne Crystallography Software (France).

	Cu^{2+} [a]	NY7	QY7	SY7	GY7
Distances (Å)	2.087	2.398	2.091	1.937	2.043
	1.807	1.555	1.444	1.526	1.500
	1.278	1.297	1.202	1.217	1.196
	1.090	-	1.048	1.014	-
	0.808	0.863	0.795	0.818	0.831

Table S3. Electronic diffraction distances from Ni^{2+} incubated fibrils. Distances from Ni^{2+} electronic diffraction pattern for each sample were obtained from electronic diffraction micrographs shown in Figure S6, obtained by TEM, using ImageJ Software (NIH, USA). [a] Reference electron diffraction pattern distances from Ni^{2+} are shown in blue and were obtained from the database of CaRIne Crystallography Software (France).

	Ni^{2+} [a]	NY7	QY7	SY7	GY7
Distances (Å)	3.524	3.484	-	2.905	-
	2.492	-	-	2.546	-
	2.035	2.105	1.908	2.092	2.108
	1.762	-	-	1.712	1.871
	1.576	-	-	1.616	1.683
	1.246	1.198	1.505	1.397	1.225
	1.062	1.052	1.085	1.063	1.058
	0.808	0.811	0.820	0.861	0.839
	0.788	0.746	0.722	-	0.712

Table S4. Electronic diffraction distances from Co^{2+} incubated fibrils. Distances from Co^{2+} electronic diffraction pattern for each sample were obtained from electronic diffraction micrographs shown in Figure S6, obtained by TEM, using ImageJ Software (NIH, USA). [a] Reference electron diffraction pattern distances from Co^{2+} are shown in blue and were obtained from the database of CaRIne Crystallography Software (France).

	Co^{2+} [a]	NY7	QY7	SY7	GY7
Distances (Å)	2.035	-	2.042	2.034	-
	1.916	1.958	1.948	1.961	1.856
	1.485	1.467	1.517	1.469	-
	1.254	1.295	1.285	1.287	1.248
	1.067	1.175	1.057	1.059	1.053
	1.049	1.063	-	-	0.912
	0.848	0.853	0.852	-	0.892
	0.804	0.773	-	0.804	-
	0.790	0.701	0.738	0.653	0.666

Table S5. Electronic diffraction distances from Zn^{2+} incubated fibrils. Distances from Zn^{2+} electronic diffraction pattern for each sample were obtained from electronic diffraction micrographs shown in Figure S6, obtained by TEM using ImageJ Software (NIH, USA). [a] Reference electron diffraction pattern distances from Zn^{2+} are shown in blue and were obtained from the database of CaRIne Crystallography Software (France).

	Zn^{2+} [a]	NY7	QY7	SY7	GY7
Distances (Å)	2.035	2.080	2.043	2.058	2.198
	1.916	1.954	-	1.957	1.731
	1.485	1.501	1.482	1.444	1.450
	1.254	1.259	1.258	1.229	1.323
	1.067	1.070	1.068	1.074	1.100
	1.049	1.044	1.038	1.044	-
	0.848	0.853	0.846	0.938	0.887
	0.804	0.816	0.803	-	-
	0.790	0.762	0.776	-	0.798

Table S6. Kinetic parameters of the fibrils' esterase activity in the presence of Cu^{2+} and Zn^{2+}. Data were fitted to Michaelis-Menten equation with GraphPad PRISM 5.0 software and kinetic parameters obtained are shown. Data correspond to the mean of 3 independent experiments (± standard deviation).

Cation	Peptide	K_M (mM)	K_{cat} x 10^{-2} (s^{-1})	K_{cat}/K_M ($M^{-1}\cdot s^{-1}$)
Cu^{2+}	NY7	3.83 ± 1.21	0.64 ± 0.10	1.68 ± 0.80
	QY7	1.71 ± 0.37	0.54 ± 0.04	3.16 ± 1.15
	SY7	1.87 ± 0.30	0.64 ± 0.03	3.42 ± 1.07
	GY7	1.37 ± 0.22	0.52 ± 0.03	3.80 ± 1.41
Zn^{2+}	NY7	2.94 ± 0.87	0.61 ± 0.09	2.07 ± 1.08
	QY7	1.35 ± 0.22	0.66 ± 0.04	4.96 ± 1.97
	SY7	1.09 ± 0.24	0.63 ± 0.05	5.78 ± 2.07
	GY7	1.07 ± 0.25	0.64 ± 0.06	5.98 ± 2.30

Table S7. Carbonic anhydrase activity of the self-assembled fibrils in the presence of Cu^{2+} and Zn^{2+}. Kinetics were followed by the decrease of absorbance at 560 nm from phenol red pH indicator. Values correspond to the differences between the initial and final absorbance values at 560 nm. Negative and positive controls are shown in blue and red, respectively.

Peptide	ΔAbs 560 nm	
	Cu^{2+}	Zn^{2+}
Buffer	-0.154	-0.215
Buffer + Metal	-0.341	-0.056
Carbonic Anhydrase (CA)	-1.047	-0.811
NY7 + Metal	-1.189	-0.832
QY7 + Metal	-0.909	-0.887
QY7	-0.231	-0.242
SY7 + Metal	-0.697	-0.747
GY7 + Metal	-0.677	-1.024

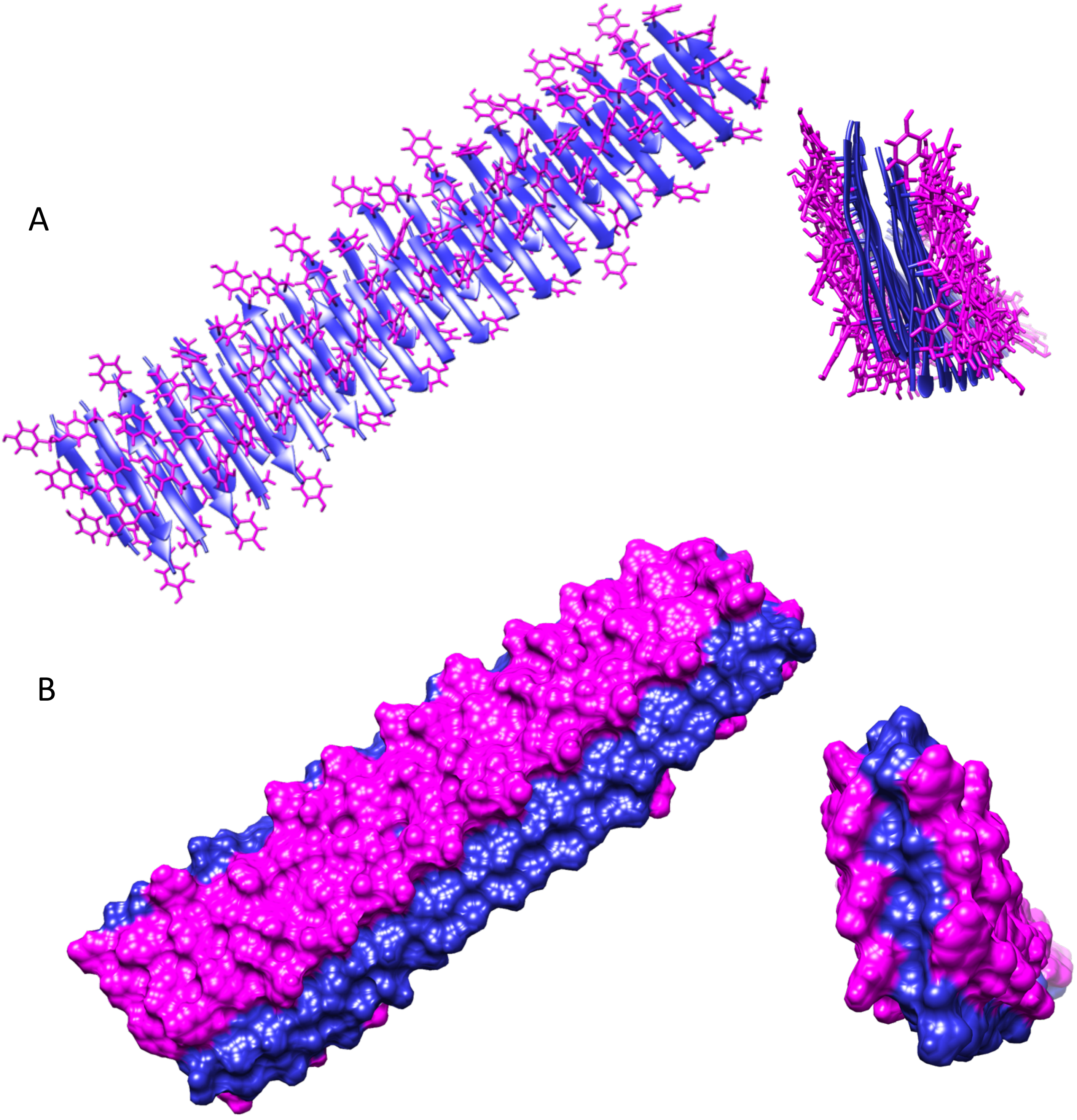

Figure S1. Model of GY7 fibrils. Structure of the most stable steric zipper of GY7 fibrils after 100 ns of Molecular Dynamics simulations as reported in [1]. The backbone is shown in blue and side chain atoms in pink. **A)** Ribbons and sticks representation. **B)** Surface representation. Left and right panels correspond to fibrils lateral and top views, respectively. Observe that all Tyr side chains face the solvent.

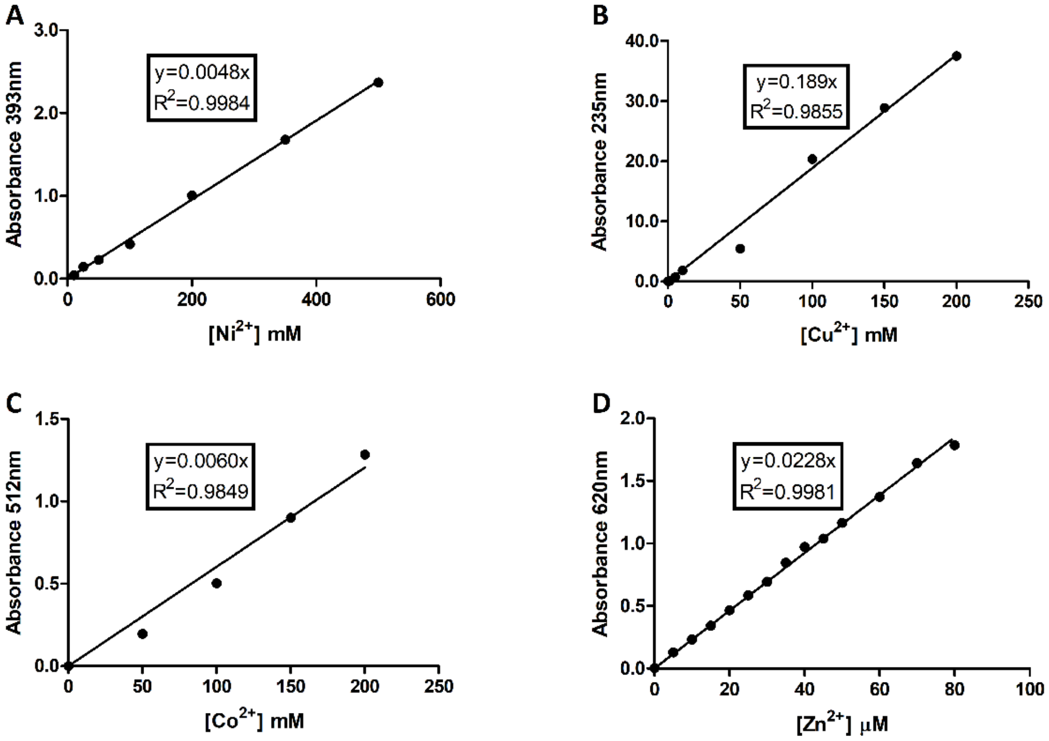

Figure S2. Experimental determination of molar extinction coefficient for divalent cations Ni^{2+}, Cu^{2+}, Co^{2+} and Zn^{2+}(Zincon complex). The experimental molar extinction coefficient (ε) for each element was obtained from the slope of the calibration curves. Calibration curves of divalent cations: **A)** Ni^{2+} (λNi^{2+} = 393 nm), **B)** Cu^{2+} (λCu^{2+} = 235 nm), **C)** Co^{2+} (λCo^{2+} = 512 nm), and **D)** Zn^{2+} in the presence of Zincon sodium salt (Zincon-Zn^{2+}) (λZincon-Zn^{2+} = 620 nm), respectively. The slope of each equation corresponds to extinction molar coefficient.

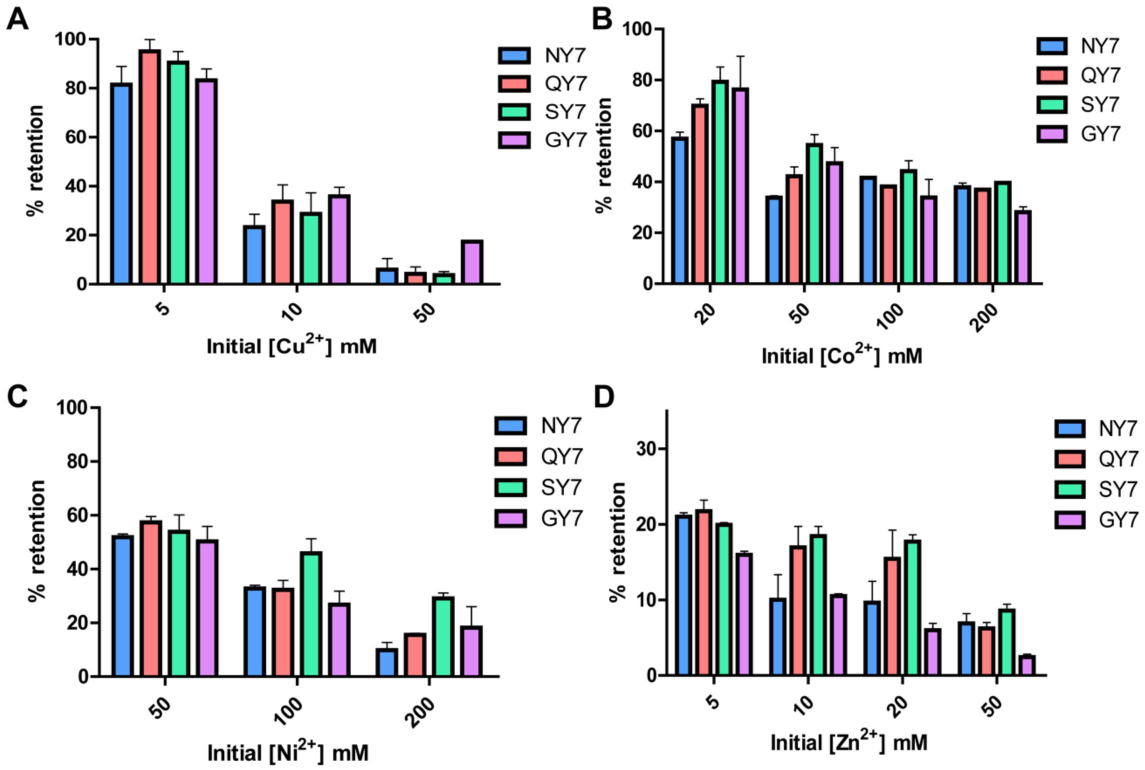

Figure S3. Fibrils retention capacity for Cu^{2+}, Co^{2+}, Ni^{2+} and Zn^{2+}. Retention percentages were determined by measuring the absorbance of NY7 (blue), QY7 (red), SY7 (green), and GY7 (purple) supernatants in the presence of each divalent cation, relative to a reference solution of each divalent cation without fibrils. **A)** Cu^{2+} incubated samples measured at 235 nm. $CuCl_2$ was added at concentrations ranging from 5 to 50 mM. **B)** Co^{2+} incubated samples measured at 512 nm. $CoCl_2$ was added at concentrations ranging from 20 to 200 mM. **C)** Ni^{2+} incubated samples measured at 393 nm. $NiSO_4$ was added at concentrations ranging from 50 to 200 mM. **D)** Zn^{2+} incubated samples, detected with Zincon sodium salt and measured at 620 nm. $ZnCl_2$ was added at concentrations ranging from 5 to 50 mM.

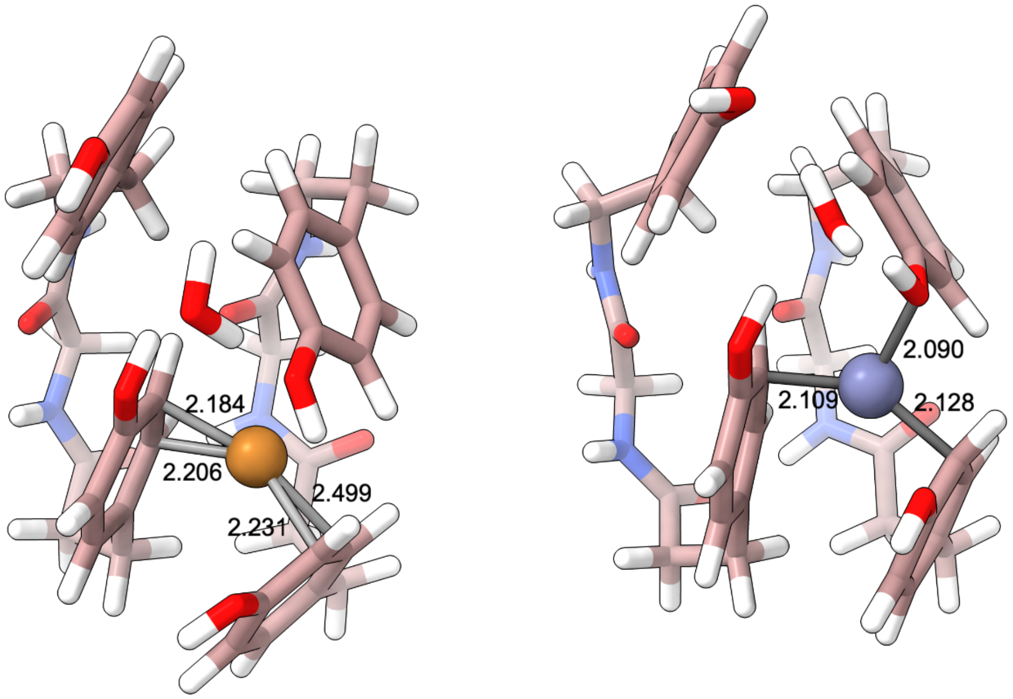

Figure S4. Optimized structures for Cu^{2+} (orange) and Zn^{2+} (blue) interacting with the π system of Tyrosines.

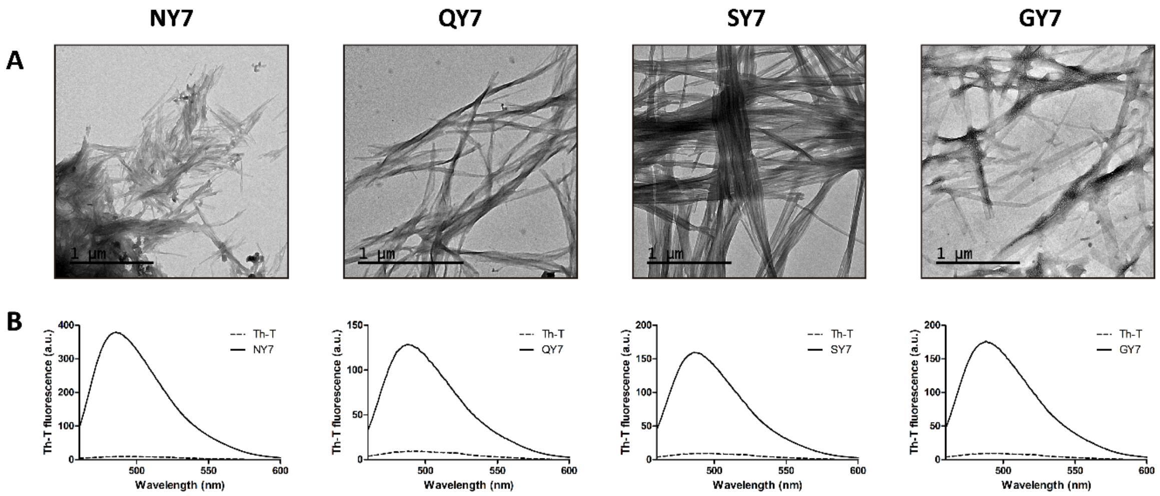

Figure S5. Characterization of NY7, QY7, SY7 and GY7 fibrils without divalent cations. A) Images corresponding to transmission electron microscopy (TEM) micrographs from fibrils upon negative staining. Scale bar corresponds to 1 µm. **B)** Graphs corresponding to Th-T fluorescence spectra in the presence of fibrils (continuous line) compared with that of Th-T without fibrils (dashed line).

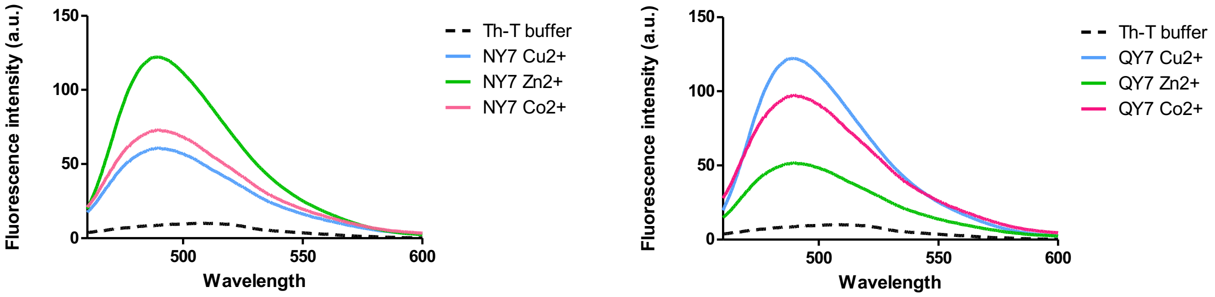

Figure S6. Fluorescence emission spectra of Th-T in the presence of NY7 and QY7 fibrils incubated with Cu^{2+}, Zn^{2+} and Co^{2+}.

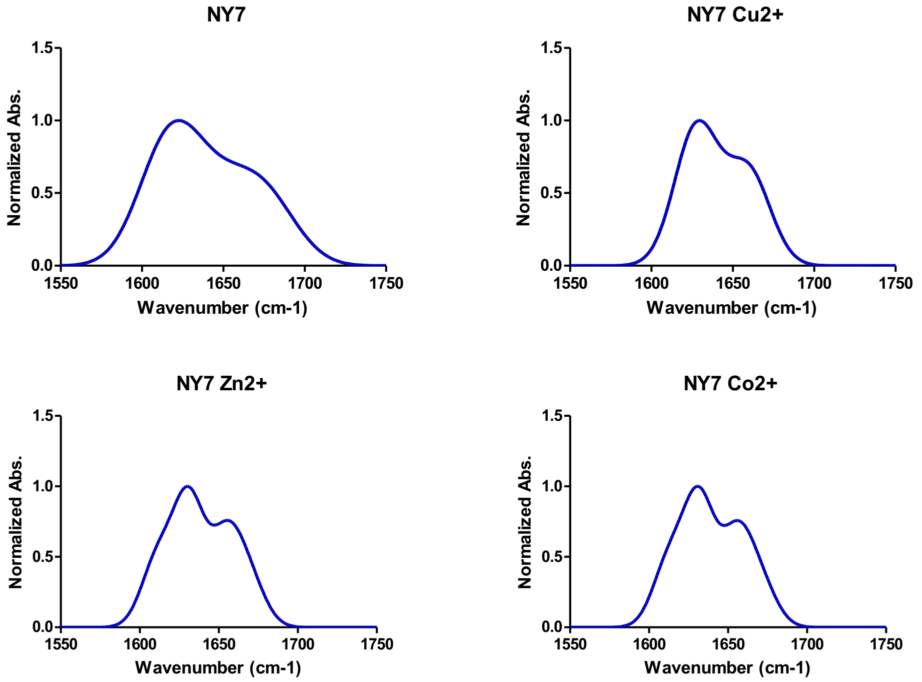

Figure S7. FT-IR absorbance spectra in the amide I region of NY7 fibrils alone or incubated with Cu^{2+}, Zn^{2+} and Co^{2+}. The major signal at 1615-1630 cm^{-1}, corresponds to β-sheets and the second band at 1655-1660 cm-1 to β-turns.

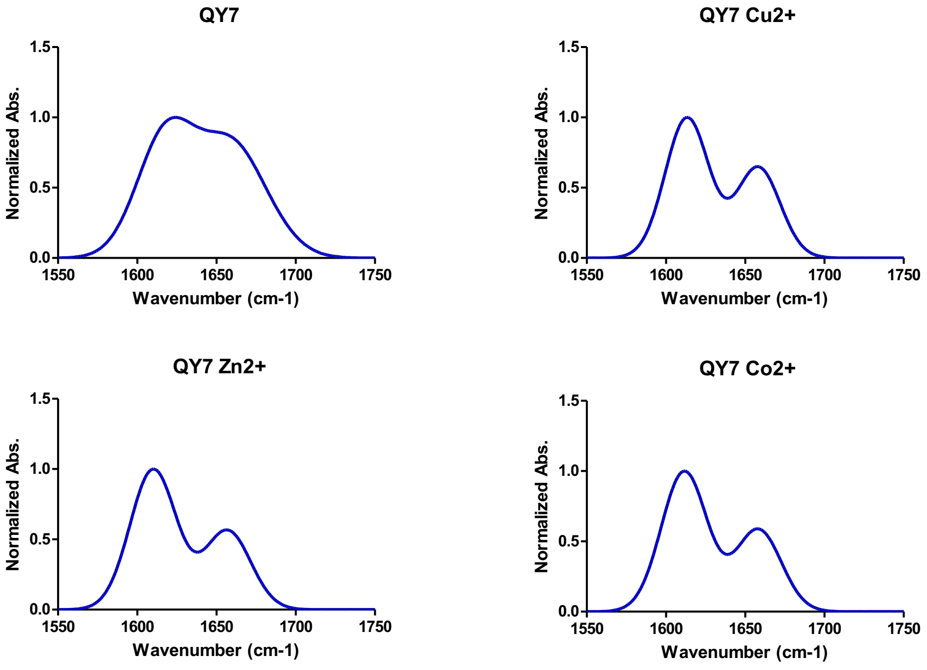

Figure S8. FT-IR absorbance spectra in the amide I region of QY7 fibrils alone or incubated with Cu^{2+}, Zn^{2+} and Co^{2+}. The major signal at 1615-1630 cm^{-1}, corresponds to β-sheets and the second band at 1655-1660 cm-1 to β-turns.

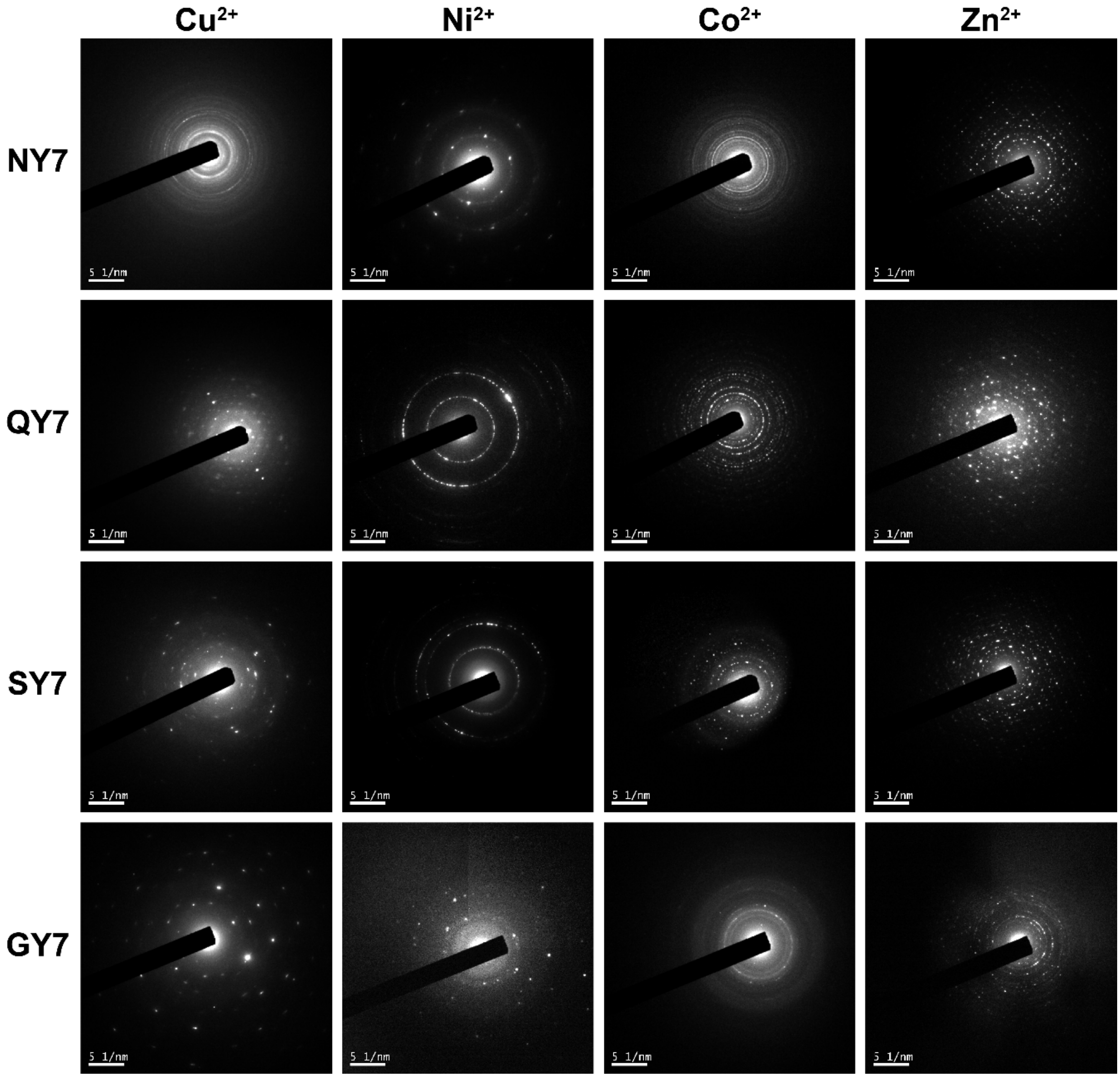

Figure S9. Electronic diffraction pattern micrographs from heptapeptides fibrils incubated with Cu^{2+}, Ni^{2+}, Co^{2+} and Zn^{2+}. Scale bars correspond to 5 nm^{-1}.

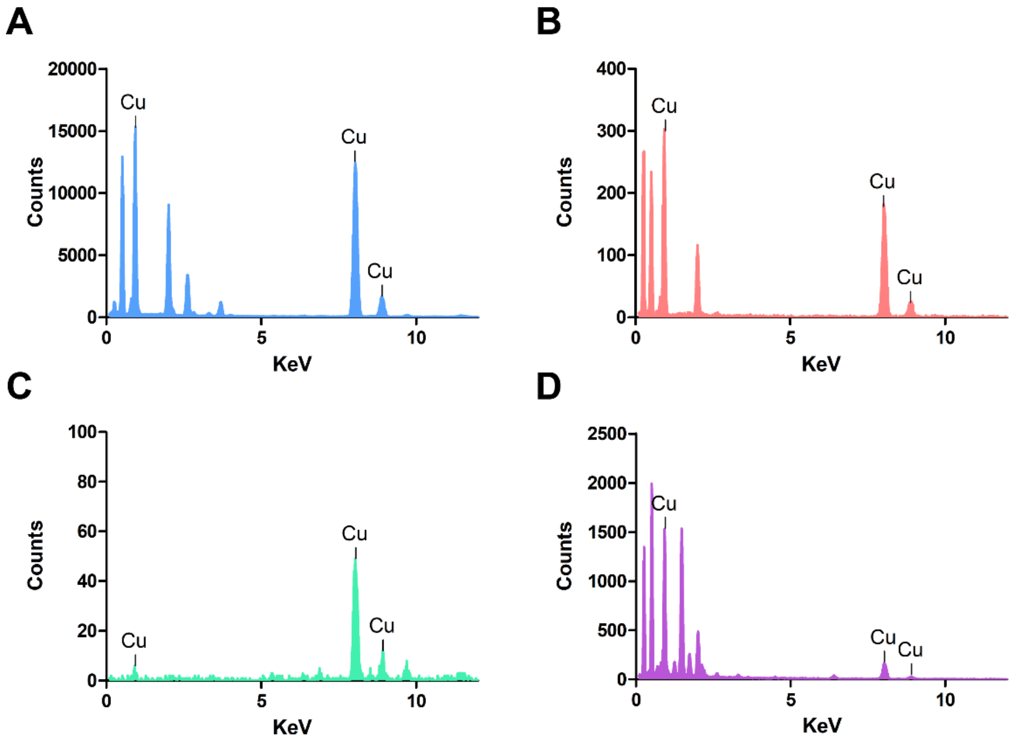

Figure S10. Electronic dispersive X-ray spectroscopy spectra from heptapeptides fibrils incubated with Cu^{2+}. EDX spectra from **A)** NY7 (blue), **B)** QY7 (red), **C)** SY7 (green) and **D)** GY7 (purple) samples incubated with Cu^{2+}.

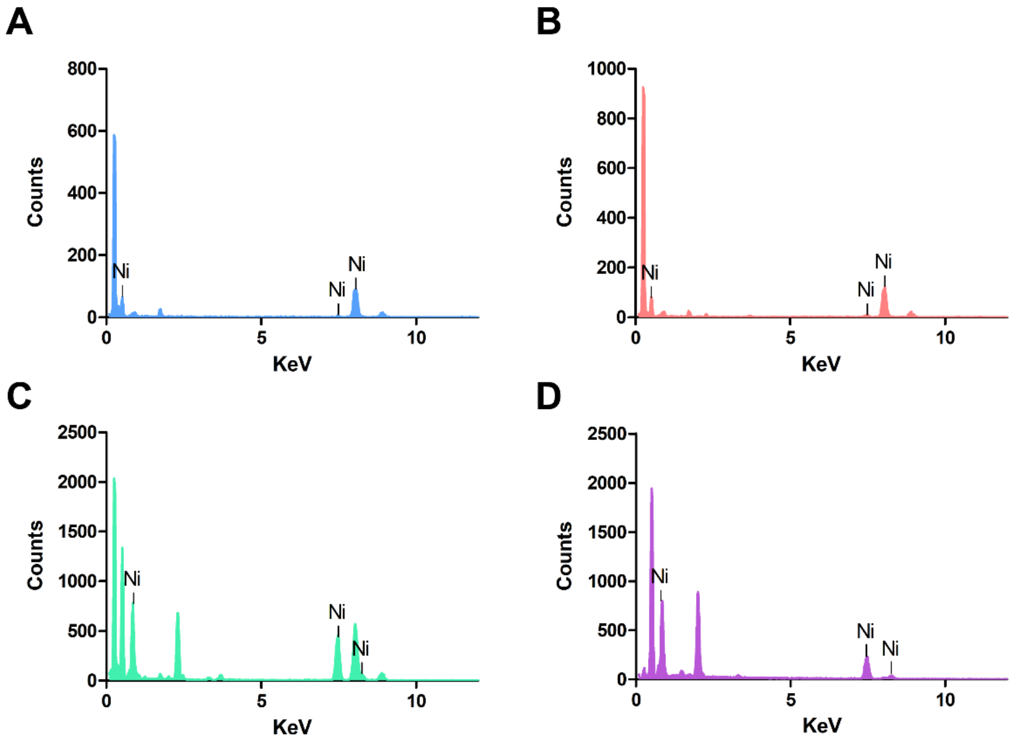

Figure S11. Electronic dispersive X-ray spectroscopy spectra from heptapeptides fibrils incubated with Ni^{2+}. EDX spectra from **A)** NY7 (blue), **B)** QY7 (red), **C)** SY7 (green) and **D)** GY7 (purple) samples incubated with Ni^{2+}.

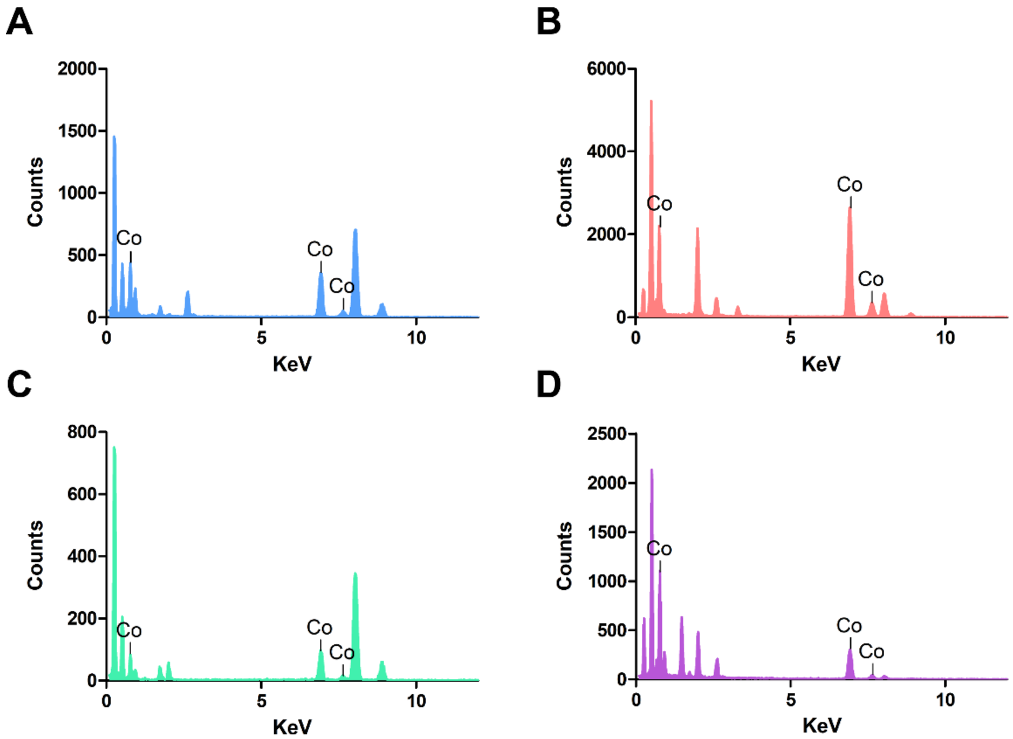

Figure S12. Electronic dispersive X-ray spectroscopy spectra from heptapeptides fibrils incubated with Co^{2+}. EDX spectra from **A)** NY7 (blue), **B)** QY7 (red), **C)** SY7 (green) and **D)** GY7 (purple) samples incubated with Co^{2+}.

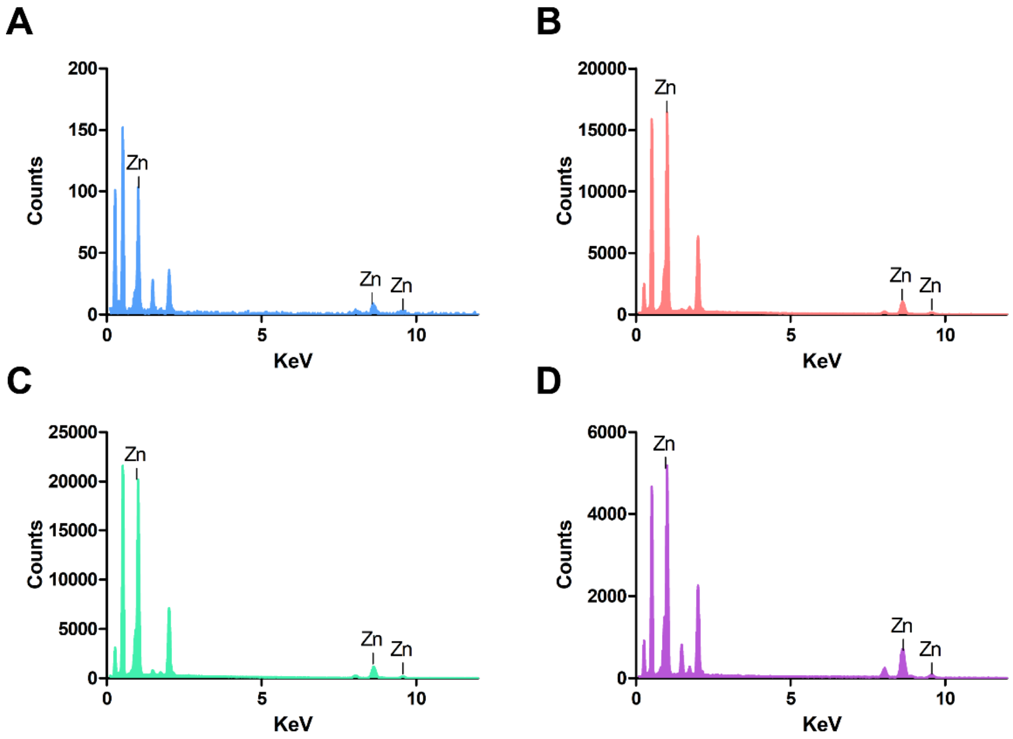

Figure S13. Electronic dispersive X-ray spectroscopy spectra from heptapeptides fibrils incubated with Zn^{2+}. EDX spectra from **A)** NY7 (blue), **B)** QY7 (red), **C)** SY7 (green) and **D)** GY7 (purple) samples incubated with Zn^{2+}.

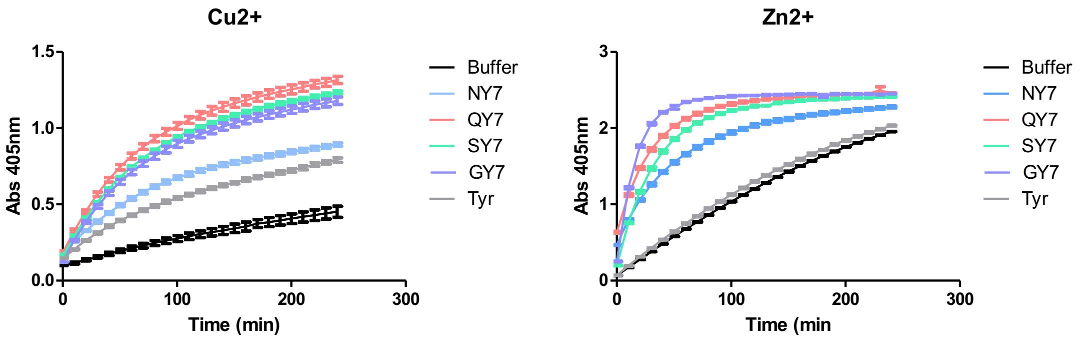

Figure S14. Fibrils esterase activity kinetics in the presence of Cu^{2+} and Zn^{2+}. Transformation of pNPA into pNP was measured along time by monitoring the increase in absorbance at 405 nm, with a peptide concentration of 100 µM. L-Tyr was used as a control.

References

1. Peccati, F.; Diaz-Caballero, M.; Navarro, S.; Rodriguez-Santiago, L.; Ventura, S.; Sodupe, M., Atomistic fibrillar architectures of polar prion-inspired heptapeptides. *Chem Sci* **2020,** *11* (48), 13143-13151.
